# Tuneable endogenous mammalian target complementation via multiplexed plasmid-based recombineering

**DOI:** 10.1038/srep17432

**Published:** 2015-11-27

**Authors:** Violeta Beltran-Sastre, Hannah Benisty, Julia Burnier, Imre Berger, Luis Serrano, Christina Kiel

**Affiliations:** 1 EMBL/CRG Systems Biology Research Unit, Centre for Genomic Regulation (CRG), Dr. Aiguader 88, 08003 Barcelona, Spain; 2 Universitat Pompeu Fabra (UPF), 08003 Barcelona, Spain; 3 European Molecular Biology Laboratory, Grenoble Outstation, B.P. 181, Grenoble, France; 4 The School of Biochemistry, University of Bristol, Clifton BS8 1TD, United Kingdom; 5 Institució Catalana de Recerca i Estudis Avançats (ICREA), Pg. Lluís Companys 23, 08010 Barcelona, Spain

## Abstract

Understanding the quantitative functional consequences of human disease mutations requires silencing of endogenous genes and expression of mutants at close to physiological levels. Changing protein levels above or below these levels is also important for system perturbation and modelling. Fast design optimization demands flexible interchangeable cassettes for endogenous gene silencing and tuneable expression. Here, we introduce ‘TEMTAC’, a multigene recombineering and delivery system for simultaneous siRNA-based knockdown and regulated mutant (or other variant) expression with different dynamic ranges. We show its applicability by confirming known phenotypic effects for selected mutations for BRAF, HRAS, and SHP2.

Recent work provided evidence that quantitative rather than qualitative differences in the proteomic inventory between different cell types and tissues are the cause of functional differences^1^,^2^,^3^,^4^,^5^,^6^,^7^. Thus, to investigate cell type-specific quantitative consequences of disease mutations or alternative spliced variants, it is of crucial importance to combine knockdown of the endogenous protein(s) with the precise regulated expression of the mutant protein(s) to the desired physiological level.

Gene silencing based on short interfering RNAs (siRNA) has been demonstrated in several organisms, including in cultured mammalian cells^8^, and works by targeting complementary messenger RNA (mRNA) thereby activating the RISC complex causing sequence-specific mRNA degradation. Short hairpin RNAs (shRNAs) can also be stably expressed downstream of polymerase III (Pol III) promoters^9^ or bidirectionally co-expressed^10^. Advanced genome editing technologies such as meganucleases, zinc finger nucleases, TALENS (protein-DNA guided), and the RNA-guided CRISPR-Cas9 and CRISPRi-dCas9 methods complement the available tools for gene silencing, each with their own merits^11^,^12^. However, despite these being powerful tools, they could modify the genome irreversibly and have other drawbacks. These may include off-target mutagenesis, although in some cases they have been significantly diminished^13^, and often cumbersome and time-consuming selection/screening to identify the appropriate clone, which is refractory to high-throughput^14^.

Plasmid-based gene delivery systems have become essential for molecular and cell biology. However, the number of available selection markers and the physical space that needs to be available to allow entry of multiple plasmids into cells are technically limiting. These problems are exacerbated by imbalanced delivery if multiple plasmids are used, resulting in heterogeneous cell populations which interferes with read-out. These impediments can be circumvented by combining independent modules containing genes of interest, regulatory elements and other desired functionalities into a single multifunctional multigene delivery plasmid. This is a viable option if the combination of the modules is sufficiently straight-forward. Addressing this challenge, we previously developed the ‘ACEMBL’ system to enable fast and flexible generation of multigene delivery constructs by an automatable technique called tandem recombineering (TR) to combine ‘Donor’ and ‘Acceptor’ plasmid modules via reversible Cre-loxP fusion *in vitro*, optionally in high-throughput by robotics^15^,^16^. We implemented ACEMBL successfully for complex expressions in prokaryotic and eukaryotic cells. However, a useful tool that would afford the means to combine efficient DNA delivery with regulated heterologous expression and efficient silencing in cell-based complementation assays remained elusive to date.

Here, we introduce TEMTAC, a novel system for **T**unable **E**ndogenous **M**ammalian **T**Arget **C**omplementation by multiplexed plasmid-based recombineering. Retaining the original ACEMBL concept, we developed this novel system which affords the means to simultaneously knockdown endogenous targets via RNA interference^8^,^9^,^10^ and express mutated versions utilizing regulated tetracycline (TET) controlled transcriptional activation^17^. Three modules are combined, one each for (i) shRNA-mediated silencing, (ii) TET-regulated expression of the mutant gene of interest (GOI) that contains silent mutations to forestall its own degradation, and (iii) integration of the complete composite plasmid, which expresses an additional YFP marker gene for identifying transfected cells by fluorescence (Fig. 1a). The three modules can be easily assembled and disassembled *in vitro* exploiting the Cre-Lox recombination reaction, which can be carried out optionally in high-throughput by robotics applying automated routines we have implemented^16^. The separate modules also work autonomously, for example to test proper functioning of each individual element prior to multigene recombineering. A homing endonuclease (HE)/BstXI based multiplication module^15^ is provided to enable engineering within each Donor or Acceptor, if several proteins, for example entire regulatory cascades, are to be complemented concomitantly. As a further option, TEMTAC also affords stable integration into cellular genomes, by providing on the Acceptor module eukaryotic resistance markers and the DNA elements required. We demonstrate the utility of TEMTAC utilizing proteins of the ErbB signalling network, HRAS, BRAF, and SHP2. By using TEMTAC, we silence the proteins of interest, express corresponding mutants, and test these for signalling phenotypes, compellingly validating our approach.

## Results

### Endogenous gene silencing module

**Figure 1:**
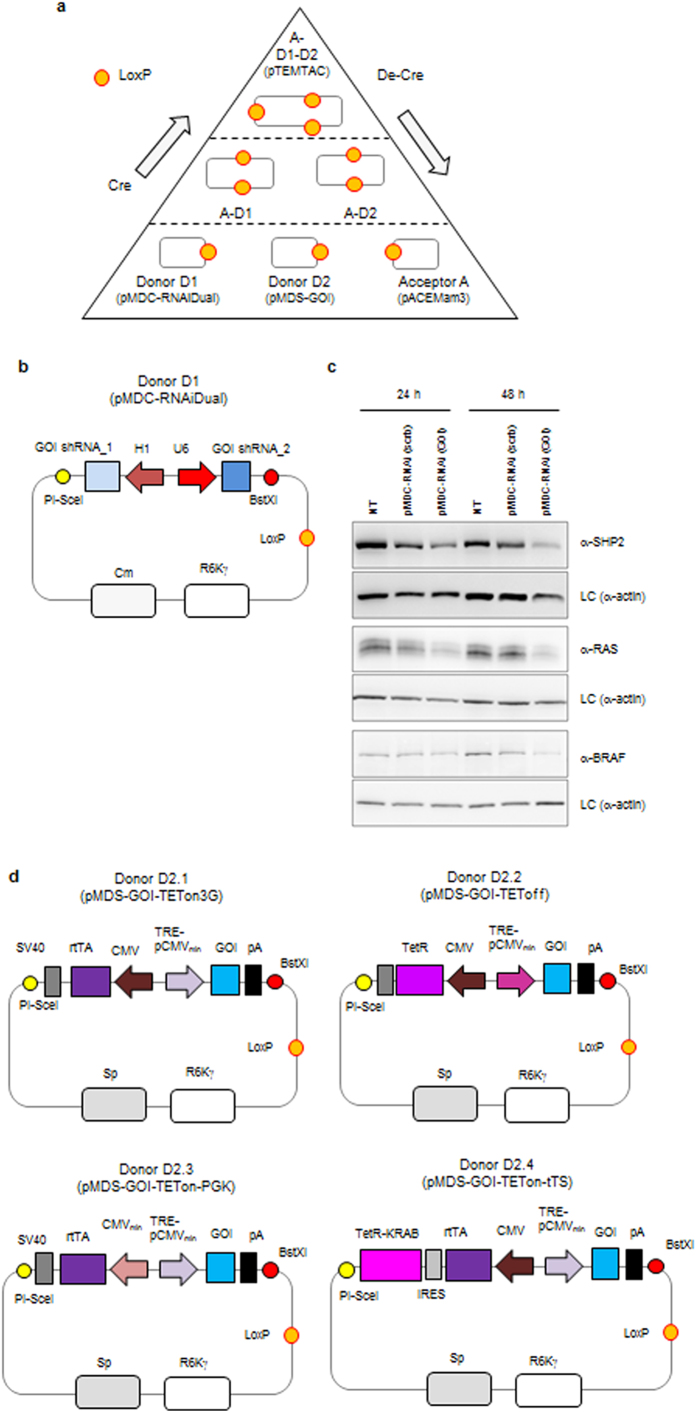
TEMTAC system components. (**a**) Cre-LoxP mediated generation of plasmid fusions is shown in a schematic view. Acceptor A plasmid module is incubated with two Donor modules, D1 and D2 in the presence of Cre recombinase. Concomitant assembly (Cre) and excision (De-Cre) reactions occur until equilibrium is reached. Acceptor-Donor (A-D1, A-D2) and Acceptor-Donor-Donor (A-D1-D2 or “pTEMTAC”) fusion plasmids co-exist with educt plasmids when equilibrium is reached. Acceptor A contains a common origin or replication (ColE1), Donors D1 and D2 contain conditional origins of replication derived from phage R6Kγ, rendering their propagation in regular cloning strains dependent on productive Cre fusion with Acceptor A. (**b**) Donor D1 (pMDC-RNAiDual) is shown in a schematic view. This Donor provides cassettes for multiple shRNA production. (**c**) shRNA-mediated downregulation of SHP2, HRAS and BRAF after transfection with a Donor D1 producing specific shRNAs. Transfected HEK293 (for HRAS and BRAF) or GH-HEK293 cells were lysed and analysed by Western blotting. (**d**) Four Donor plasmid variants D2.1 to D2.4 are shown schematically, which realize four distinct dynamic ranges of exogenous protein expression. *Abbreviations*: Cre, Cre recombinase enzyme; LoxP, imperfect inverted repeat recognized by Cre; GOI, gene of interest; A-D1, fusion of Acceptor A with Donor D1; A-D2, fusion of Acceptor A with Donor D2 (or variants); A-D1-D2, complete fusion of Acceptor A with Donors D1 and D2 (or variants); shRNA, small hairpin RNA sequence; I-SceI, PI-PspI and PI-SceI are homing endonucleases; H1, U6, CMV and CAG are common mammalian active promoters; pA and SV40 are common poly-adenylation signals; TRE-pCMVmin, tetracycline response element with minimal CMV promotor; rtTA, tetracycline transactivator with (random) mutagenesis derive Tet repressor part of the transactivator gene; Sp, Cm, Hygr and Zeo denote resistance marker genes for spectinomycin, chloramphenicol, hygromycin and zeocin, respectively; YFP, yellow fluorescence protein; TetR, tet repressor gene; TetR-KRAB, tetracycline-controlled hybrid protein of TetR with the KRAB silencing domain of human Kid1; IRES, internal ribosome entry site; Frt, FLP recognition target; ColE1, common colicin E1 derived replication origin; R6Kγ, conditional origin derived from R6Kγ phage.

Donor D1 (pMDC-RNAiDual) contains the silencing cassette for knocking down the endogenous gene by RNA interference (Fig. 1b). In one Donor D1, two shRNAs can be bi-directionally co-expressed downstream of H1 and U6 Pol III promoters. This allows the testing of the silencing effects of multiple shRNA hairpin sequences. In addition, previous work has shown that silencing effects are often additive and that combination therefore increases knockdown efficiency, without cumulative off-target effects^18^. We tested the silencing effect of the independent Donor D1 for three proteins. For BRAF and SHP2, we inserted two published siRNA sequences^19^,^20^,^21^,^22^ into Donor D1. In the case of RAS we cloned new shRNAs which we designed using online software (http://www.invivogen.com/sirna-wizard). Donor D1 containing the shRNAs was transiently transfected in HEK293 or HeLa cells. After 24 and 48 hours, cells were lysed and endogenous protein levels detected by Western blotting, real time PCR, and deep sequencing. We observed the best silencing effect 48 h post transfection and no (or small) down-regulation using random (‘scrambled’ scrb) shRNAs as a control (Fig. 1c and Supplementary Figs. 1 to 4). Incomplete silencing will depend on the efficiency/specificity of the respective shRNA’s used. A further aspect is that transfection is transient and the transfection efficiency is small than 80-90% in most cell lines.

### Inducible exogenous mutant expression module

Achieving expression levels that are close to endogenous levels is a crucial prerequisite for analysing the physiological effect of mutations. This is particularly important when studying signalling proteins because of their frequent involvement in downstream activation and feedback loops, which often function at very precise protein levels that need to be maintained meticulously. A major problem in many TET regulated systems is basal expression (without the inducer being present), especially for proteins that are present at markedly low abundance in cells. Furthermore, depending on the protein of interest, different dynamic expression ranges of mutants would be highly desirable to properly recapitulate physiological events. To address this, we created as Donor D2 variants four different TET-related expression system versions with defined properties (Donor D2.1 to D2.4, Fig. 1d and Supplementary Fig. 5).

- Donor D2.1 (pMDS-GOI-TETon3G), constitutively expresses the TETon3G transactivator (Clontech, rtTA), a fusion protein of a Tet repressor (*E. coli*) mutant and three VP16 (transcription activation domain of herpes simplex virus). In the presence of the inducer doxycycline (dox), the TETon3G-dox complex binds to the TRE minimal promoter (TRE-pMCVmin) and induces the expression of the gene of interest (GOI).
- Donor D2.2 (pMDS-GOI-TEToff) is based on the TEToff advanced inducible expression system (Clontech). This vector expresses the TEToff advanced transactivator (TetR) and also contains a TRE-based module that expresses high levels of the GOI in the absence of dox.
- Donor D2.3 (pMDS-GOI-TETon-PGK), is a modification of Donor D2.1, where the expression of the rtTA is downstream of a minimal phosphoglycerate kinase (PGK) promoter (reduced basal expression).
- Donor D2.4 (pMDS-GOI-TETon-tTS) is a modification of TETon to reduce basal expression levels. This is achieved by adding an internal ribosome entry site (IRES) and a tTS (Tetracycline-controlled transcriptional silencer, Clontech), which is a fusion of the TetR and the KRAB-AB silencing domain of the Kid-1 protein, after the transactivator (rtTA). As tTS competes with rtTA by binding specifically to the TRE, it suppresses the transcription in the absence of dox.

**Figure 2:**
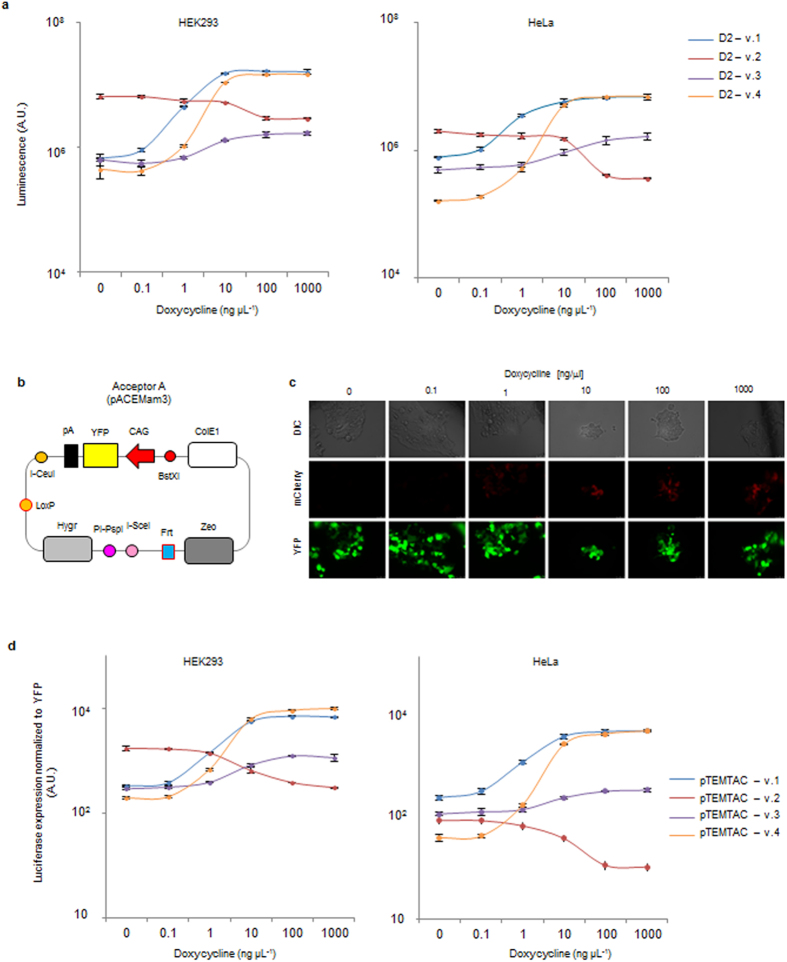
Luciferase expression using different donor plasmids and using the assembled pTEMTAC. **(a)** Luciferase expression in HEK293 and Hela cells from the different Donor D2 versions shown in Fig. 1 panel **d**. Averages and standard deviations from three biological replicates are shown. (**b**) Schematic view of Acceptor A (pAceMam3) plasmid. (**c**) Expression of mCherry from a complete A-D1-D2 fusion plasmid in HEK293 cells is shown, using different doxycycline concentrations for induction. Transfection efficiency was visualized by monitoring YFP fluorescence. (**d**) Luciferase expression in HEK293 and Hela cells from the different pTEMTAC plasmid. Averages and standard deviations from three biological replicates are shown. *Abbreviations*: see Figure legend 1.

To compare their performance, we inserted the luciferase gene into D2.1 to D2.4 and monitored luciferase expression at increasing doxycycline concentrations in HEK293 and HeLa cells (Fig. 2a and Supplementary Fig. 6). In our experiments, Donor D2.4 is characterized by the lowest basal expression and higher dynamic range. Donor D2.3 exhibited a narrow dynamic range and medium basal levels, while D2.1 is intermediate in between D2.3 and D2.4. Donor D2.3 could be interesting when aiming for an expression level in the exponential expression range of D2.4: in this case it might be difficult to tune the right levels of dox for D2.4, but this should be doable if D2.3 is used. D2.2 has a high basal expression and can be efficiently repressed. Thus, TEMTAC offers four defined expression modalities that cover the requirements for the large majority of genes to be expressed.

### Integrator module

The final component of TEMTAC is Acceptor A, which provides the integrator cassette that receives the elements from both the silencing and inducible expression modules (Fig. 2b). Acceptor A contains the elements required for Donor integration, construct selection, and transfection visualization by monitoring expression of yellow fluorescent protein (YFP). In addition to transient multigene expression by transfection, Acceptor A can also be used to generate stable cell lines, as it contains the elements required for stable integration of multigene constructions. To this end, two rare homing endonuclease sites, I-SceI and PI-PspI, are included in the Acceptor, between two eukaryotic drug selection markers for efficient linearization with these extremely rare cutters. The two resistance markers are flanking the multigene construction that would result from Cre-mediated Donor D1 and D2 incorporation into Acceptor A, to ensure integration of the complete multigene construction in the resulting stably expressing cell lines by exposure to both drugs simultaneously during selection. Moreover, Acceptor A also contains the flippase recognition target (Frt) site for stable integration into commercially available flippase (Flp)-expressing cells (i.e. Flp-In-293, Invitrogen) if desired. We tested Acceptor A by Cre-LoxP fusing with Donor D2.1-mCherry (containing mCherry as GOI). We observed both a high transfection efficiency transfection (monitored by YFP expression) and moreover also proper TET-regulated expression of the mCherry protein (Fig. 2c) and of luciferase (Fig. 2d and Supplementary Fig. 6).

### Biological application

**Figure 3:**
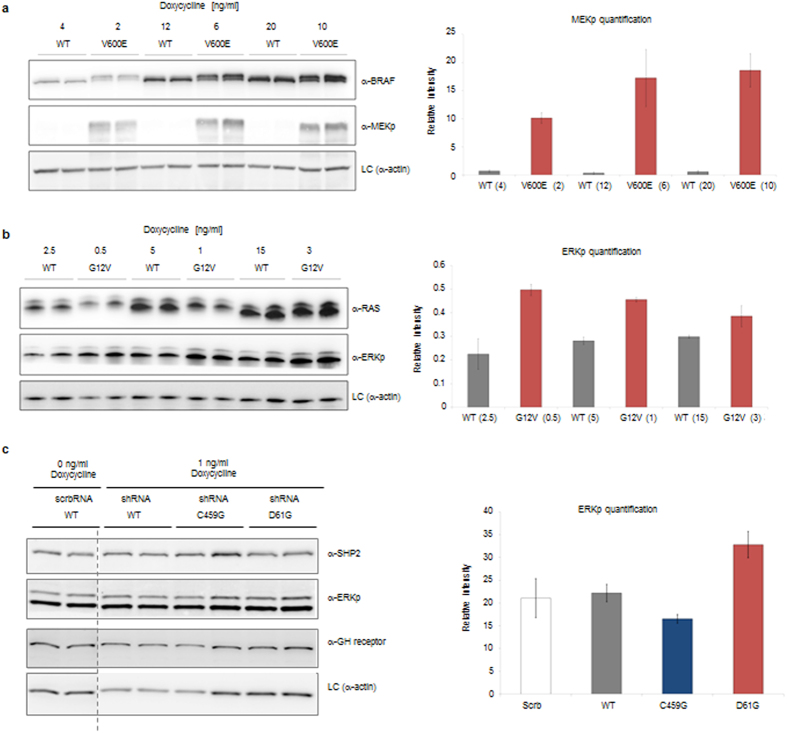
Highly efficient wild-type down**-**regulation and concomitant mutant complementation for BRAF, HRAS and SHP2 proteins by TEMTAC. (**a**) Expression and phenotype analysis of BRAF V600E and WT in HEK293 cells is shown. Left panel: Western blot results using specific antibodies as indicated. Right panel: MEKp quantification after intensity analysis using ImageJ. (**b**) Expression and phenotype analysis of HRAS G12V and WT in HEK293 cells. Left panel: Western blot results using antibodies as indicated. Right panel: MEKp quantification after intensity analysis using ImageJ. (**c**) Expression and phenotype analysis of SHP2 C459G, D61G and WT in GH-HEK293 cells (HEK293 cells stably expressing the growth hormone receptor). Left panel: Western blot results using antibodies as indicated. Right panel: ERKp quantification after intensity analysis using ImageJ. The dashed line indicates that the blot has been cropped. The full blot is provided in Supplementary Figure S11.

We next tested our TEMTAC system with the three oncogenic proteins SHP2, BRAF and HRAS. Similar as observed for luciferase assays, the three target genes could be expressed in a doxycycline-dependent manner to very high levels (Supplementary Figure S7 and S8). In all cases, we silent-mutated the GOI to be expressed exogenously, by altering the nucleotide sequence at the 3’prime end of the positions where the two shRNAs bind, to prevent the induction of the degradation machinery on the exogenous mRNA. We first tested the combined down**-**regulation of endogenous BRAF with exogenous complementation by a BRAF V600E cancer mutation using a Donor D2.4 variant (pMDS-GOI-TETon- tTS) in HEK293 cells grown in serum-containing medium. Exogenous expression of this oncogenic mutation tends to be higher as compared to wildtype (WT) (C. Kiel *et al.* submitted, 2015). To rectify this, we took advantage of our TET-regulated system to tune the expression levels of WT and V600E to be close to identical (4 ng/ml of doxycycline to express WT and 2 ng/ml of doxycycline to express V600E; Fig. 3a and Supplementary Fig. 9). Higher, but also similar expression levels were obtained by using ratios of 12(WT):20(V600E) and 6:10 ng/ml doxycycline, respectively. When comparing similar protein expression levels, we found in all cases much higher MEK (gene IDs MAP2K1 and MAP2K2) phosphorylation levels, which characterizes this oncogenic mutation^20^. Secondly, we expressed an oncogenic mutant of HRAS (G12V) using donor D2.1 (pMDS-GOI-TETon) in HEK293 cells. Again, higher concentrations of doxycycline were needed for the WT construct to result in similar expression levels as for HRAS (Fig. 3b and Supplementary Fig. 10). As expected, the HRAS G12V mutation resulted in higher ERK (gene IDs MAPK3 and MAPK1) phosphorylation levels. Moreover, we also tested both a phosphatase dead (C459G) and an activating (Noonan syndrome-causing, D61G) mutation of SHP2 (gene ID PTPN11) using Donor D2.1 (pMDS-GOI-TETon-3G) in HEK293 cells that stably express the growth hormone (GH) receptor^23^. SHP2 WT and mutant expression levels were similar at the same doxycycline concentrations. At 24 h after transfection, GH-HEK293 cells were induced with 1 ng/ml doxycycline in serum-free media for 24 hours. Cells were stimulated for 15 min with growth hormone, lysed in SDS buffer, and analysed by Western blotting (Fig. 3c and Supplementary Figure S11). Corroborating previous observations^24^, we found reduced ERK phosphorylation with the phosphatase dead (C459G mutation) and increased ERK signalling with the D61G mutation.

## Conclusions

We created and compellingly validated TEMTAC, a novel, highly efficient system for tuneable endogenous mammalian target complementation. TEMTAC exploits multiplexed plasmid-based recombineering, relying on automatable routines for heterologous DNA insertion and Cre-LoxP mediated plasmid fusion in custom-designed Acceptor and Donor plasmid modules, compatible with robotics and high-throughput. TEMTAC enables production of multiple different shRNAs for efficient knockdown of endogenous proteins combined with simultaneous, highly regulated expression of protein variants such as exogenous mutants. Tuning of exogenous proteins to the desired physiological level is achieved in TEMTAC by provision of several different TET-inducible promotor systems covering a large dynamic range. Moreover, it allows for testing hypotheses on how quantitative protein level alterations may impact specific cellular responses. Complex multicomponent signalling systems and even complete metabolic pathways can be analysed by TEMTAC in this way. Furthermore, cancer phenotypes, in which a complete signal cascade is often mutated, can be investigated by TEMTAC, for instance by generating arrays of variants of cascade components that approximate the malignant mutant state.

Our system is by no means restricted to mutation analysis. TEMTAC can also be used for other applications, such as to express alternative spliced isoforms to reveal their cellular function. Likewise, protein variants such as fusion proteins can be substituted for wild-type, fine-tuned at endogenous levels for subsequent comprehensive functional analysis. Tags for efficient tandem affinity purification or fusion proteins of biotin ligase can be included to detect specific endogenous signalling complexes and their dynamic assembly. Moreover, genetic elements for viral encapsulation can be incorporated in TEMTAC, for infecting primary cells to dissect mechanisms that control cell fate. A modified version of the Acceptor can be used in combination with the PiggyBac system for generating stable cell lines^25^ (Supplementary Figure S12). Furthermore, the presence of the fluorescent marker YFP enables cells to be efficiently sorted by FACS for subsequent analysis of transfected cells at the single-cell level. We anticipate that a wide range of applications will benefit from TEMTAC for genotype-phenotype analysis in mammalian systems, including genome-wide characterization of alternative splice variants, small nucleotide polymorphisms, mutations under various physiological-, developmental- and cell type- specific conditions in health and disease states.

## Methods

### TEMTAC plasmid design and preparation

Plasmids Donor D1 (pMDC-RNAiDual) and Donor D2 (pMDS-GOI) were created based on the original plasmids of the ACEMBL system for protein complex expression in prokaryotic hosts^15^. Briefly, origins of replication, resistance markers, the loxP sequence, the multiplication module consisting of matching homing endonuclease site and BstXI were retained from the original ACEMBL Donors and the former protein expression cassettes exchanged by the novel production modules for producing shRNAs (D1) or the protein variant of interest (D2), respectively. These novel modules were designed based on commonly used mammalian active expression cassettes for shRNA or genes of interest and synthesized by a commercial supplier (GenScript Corporation, Piscataway, NJ USA). Synthetic fragments were inserted into the DNA plasmid backbones using standard restriction/ligation cloning. In Donor D1 (pMDC-RNAiDual), shRNAs are bidirectionally co-expressed using specific polymerase III promoters H1 and U6, respectively, as previously described^24^. A series of Donor D2 plasmids was designed to enable tunable expression by adapting commercially available tetracycline-dependent induction (TET) modules (Clontech laboratories, Mountain View, CA USA). Thus, Donor D2.1 (pMDS-GOI-TETon3G) comprises a TET module derived from Tet-On 3G; Donor D2.2 (pMDS-GOI-TEToff) a TEToff module; Donor D2.3 (pMDS-GOI-TETon-PGK) is derived from D2.1, however with the promotor for rtTA exchanged to a minimal promotor (PGK); Donor D2.4 (pMDS-GOI-TETon-tTS) is derived from D2.1 however comprises a transcriptional silencer (Tet-tTS).

Plasmid Acceptor A was designed *de novo* from scratch. All DNA elements (YFP expression cassette, resistance markers, multiplication module, Frt site and homing endonuclease sites for linearization, see Fig. 1) were synthesized (Genscript) and combined by sequence and ligation independent cloning^26^.

All plasmids were verified by DNA sequencing (GATC Biotech, Cologne, Germany). Sequences are provided in the Supplementary Information.

### DNA manipulation

shRNAs and genes of interest (GOI) were inserted into the TEMTAC system using standard restriction-ligation cloning methods or, alternatively, by Gibson cloning^27^. Cre recombinase reactions were carried out according to the manufacturer’s recommendation (New England Biolabs, Ipswich, MA USA). Single amino acid mutations (for HRAS, SHP2, and BRAF) were introduced with the QuikChange site-directed mutagenesis kit (Stratagene, Santa Clara CA, USA) using Donor D2-based constructs as template.

### Cell culture methods

HEK293, HeLa and GH-HEK293 cells were cultured in Dulbecco’s modified Eagle’s medium (Gibco Life Technologies, Grand Island, NY, USA) supplemented with L-glutamine and 10% (v/v) heat-inactivated TET-free fetal calf serum (Clontech ref. 631106) (here called normal growth medium).

### Luciferase assay

HEK293 or HeLa cells were seeded in 96-well-plates (Thermo Scientific 165306) and grown to 60% confluency in normal growth medium. Cells were transfected with 40 ng of the four different Donor D2 plasmids containing luciferase as the GOI (Donor D2.1-D2.4) or with the fully Cre-assembled (A-D1-D2) pTEMTAC composite plasmid (containing siRNA scramble) using lipofectamine 2000 (Invitrogen, Life Technologies). 24 hours after transfection, cells were supplemented with indicated amounts of doxycycline (Sigma-Aldrich). 24 hour later the luciferase assay was performed according to the manufactures instructions (Promega Corporation, Fitchburg WI, USA).

### Microscopy

HEK293 cells were seeded on 6 well plates and grown to 60% confluency in normal growth medium. Cells were transfected with 2.5 μg of the fully Cre-assembled A-D1-D2 composite plasmid containing mCherry as the GOI (containing siRNA scramble) using lipofectamine 2000 (Invitrogen). 24 hours after transfection, cells were supplemented with indicated amounts of doxycycline (Sigma-Aldrich, Carlsbad, CA USA). 24 h later YFP and mCherry fluorescence was imaged on an inverted DMI-6000 Leica wide-field fluorescent microscope equipped with a Leica DFC 350FX camera with a 20× objective.

### Transfection, growth hormone stimulation, and cell lysis for SHP2 WT and mutants

GH-HEK293 cells (HEK293 cells stably expressing the growth hormone receptor; gift from Armelle Yart lab) were seeded on 6-cm dishes in normal growth medium, grown to 60% confluency, and transfected with 2.5 μg of pTEMTAC (A-D1-D2) plasmid (containing SHP2 WT or mutants as GOI and the corresponding RNAiDual) or, alternatively, pMDC-RNAiDual (shRNA against SHP2 or scramble shRNA) plasmid, using lipofectamine 2000 (Invitrogen) according to the manufacturer’s instructions. 24 hours after transfection, cells were supplemented with indicated amounts of doxycycline (Sigma-Aldrich) and incubated in minimal medium (DMEM plus 2 mM glutamine and 1% serum). 24 hours after transfection, cells were stimulated with 125 ng/ml growth hormone for 10 minutes, washed with PBS and resuspended in 200 μl of lysis buffer [0.1% SDS, 25 mM Tris (pH 7.8), 1:1000 protease inhibitor cocktail 1 and 2 (Sigma-Aldrich)]. Lysed cells were analysed by western blot.

### Transfection and cell lysis for HRAS and BRAF WT and mutants

HEK293 were seeded on 6-cm dishes in normal growth medium, grown to 60% confluency, and transfected with 2.5 μg of pTEMTAC (containing BRAF/HRAS WT or V600E/G12V as GOI) or pMDC-RNAiDual (shRNA against BRAF/HRAS or scramble shRNA) plasmid, using lipofectamine 2000 (Invitrogen) according to the manufacturer’s instructions. 48 hours after transfection, cells were supplemented with indicated amounts of doxycycline (Sigma-Aldrich) and incubated in normal growth medium. 24 hours after transfection, cells were washed with PBS and resuspended in 200 μl of lysis buffer [0.1% SDS, 25 mM Tris (pH 7.8), 1:1000 protease inhibitor cocktail 1 and 2 (Sigma)]. Lysed cells were analysed by western blot.

### Western blot

Cell lysates were loaded on SDS gels and separated by electrophoresis; gels were then transferred onto nitrocellulose membrane using the iBlot system (Invitrogen), and the blots were incubated 1 h at room temperature in TBS, Tween 0.1%+5% milk. The primary antibody was incubated at 4 °C overnight (1:1000 dilution), and the HRP-coupled secondary antibody (1:10 000 dilution) was incubated 1 h at room temperature, both in TBS Tween 0.1% + 0.5% milk. Blots were developed using high sensitivity ECL reagent (Thermo) and visualized using the Fujifilm LAS-3000 developer. Bands were analyzed using ImageJ. The following antibodies were used for Western blotting: SHP2 (Cell Signaling Technologies Inc, Danvers MA, USA, #3752), BRAF (SIGMA, HPA001328), total RAS (Abcam, Cambridge UK; ab52939), growth hormone receptor (Abcam, ab65304), phospho-ERK Thr202/Tyr204 (Cell Signaling Technologies Inc, #9101), phospho-MEK Ser217 and Ser221 (Cell Signaling, #9121) and β-actin (Thermo Fischer Scientific, Waltham MA USA, MA5-15739).

### Real time PCR

Cells were grown and transfected as before. RNA was isolated using miRNeasy Mini Kit (Qiagen) following the manufactures protocol including the optional DNase digestion step. PCR reactions were prepared using qPCR mix (Power SYBR^®^ Green RNA-to-C_T_^TM^ 1-Step Kit, Applied Biosystems), 0.15 μM of forward and reverse primers and 60 ng of RNA following the manufactures instructions. For PCR the cycling parameters were (i) reverse transcription (48 °C, 30 min), (ii) activation of polymerase and initial denaturation (95 °C, 10 min), (iii) 40 cycles of denaturation (95 °C, 15 sec), annealing, extension and read fluorescence (60 °C, 1 min), (iv) final hold (4 °C). The following primers were used. For endogenous BRAF 5′ATCCCAGAGTGCTGTGCTGT3′ (forward) and 5′TCTCCAACACTTCCACATGC3′ (reverse), for exogenous BRAF 5′AATGTTGCGCCGTTTACAG3′ (forward) and 5′TCTCCAACACTTCCACATGC3′ (reverse), for endogenous HRAS 5′TGCCATCAACAACACCAAGT3′(forward) and 5′ACGTCATCCGAGTCCTTCAC3′ (reverse), for exogenous HRAS 5′GAGGGCTTCCTGTGTGTCTT3′ (forward) and 5′TCTTTGACGCGCTTAATTTG3′ (reverse), for endogenous SHP2 5′CTTGTACTCCAACGCCACCC3′ (forward) and 5′CTGTGCTGAAGTTTTGGCAGG3′ (reverse) and for total SHP2 5′GGTGTGGAGGCAGAAAACCT3′ (forward) and 5′TTGATGTGGGTGACAGCTCC3′(reverse).

### Amplicon sequencing/MiSeq

RNA was extracted as described for real time PCR. cDNA synthesis was done using SuperScript^TM^ II RT (Invitrogen) with random oligonucleotides. In the first PCR all target genes (BRAF, HRAS, SHP2) were amplified for 5 cycles using Phusion DNA polymerase (New England Biolabs) with the following degenerated adaptor primers: for BRAF (region of V600E)

5′CCCTACACGACGCTCTTCCGATCTCTTCATGAAGACCTCACAG3′

5′CCCTACACGACGCTCTTCCGATCTaCTTCATGAAGACCTCACAG3′

5′CCCTACACGACGCTCTTCCGATCTgaCTTCATGAAGACCTCACAG3′

5′CCCTACACGACGCTCTTCCGATCTcgtCTTCATGAAGACCTCACAG3′

5′CCCTACACGACGCTCTTCCGATCTagatCTTCATGAAGACCTCACAG3′

(forward) and

5′TTCAGACGTGTGCTCTTCCGATCTCTGTTCAAACTGATGGGACC3′

5′TTCAGACGTGTGCTCTTCCGATCTaCTGTTCAAACTGATGGGACC3′

5′TTCAGACGTGTGCTCTTCCGATCTgaCTGTTCAAACTGATGGGACC3′

5′TTCAGACGTGTGCTCTTCCGATCTcgtCTGTTCAAACTGATGGGACC3′

5′TTCAGACGTGTGCTCTTCCGATCTagatCTGTTCAAACTGATGGGACC3′

(reverse), for HRAS (region around G12V)

5′CCCTACACGACGCTCTTCCGATCTGACGGAATATAAGCTGGTGG3′

5′CCCTACACGACGCTCTTCCGATCTaGACGGAATATAAGCTGGTGG3′

5′CCCTACACGACGCTCTTCCGATCTcaGACGGAATATAAGCTGGTGG3′

5′CCCTACACGACGCTCTTCCGATCTgctGACGGAATATAAGCTGGTGG3′

5′CCCTACACGACGCTCTTCCGATCTatcaGACGGAATATAAGCTGGTGG3′

(forward) and

5′TTCAGACGTGTGCTCTTCCGATCTGTCGTATTCGTCCACAAAGTG3′

5′TTCAGACGTGTGCTCTTCCGATCTaGTCGTATTCGTCCACAAAGTG3′

5′TTCAGACGTGTGCTCTTCCGATCTcaGTCGTATTCGTCCACAAAGTG3′

5′TTCAGACGTGTGCTCTTCCGATCTatcGTCGTATTCGTCCACAAAGTG3′

5′TTCAGACGTGTGCTCTTCCGATCTatcaGTCGTATTCGTCCACAAAGTG3′

(reverse), for SHP2 (region around D61G)

5′CCCTACACGACGCTCTTCCGATCTAGTAAAAGTAACCCTGGAGAC3′

5′CCCTACACGACGCTCTTCCGATCTaAGTAAAAGTAACCCTGGAGAC3′

5′CCCTACACGACGCTCTTCCGATCTgaAGTAAAAGTAACCCTGGAGAC3′

5′CCCTACACGACGCTCTTCCGATCTtgaAGTAAAAGTAACCCTGGAGAC3′

5′CCCTACACGACGCTCTTCCGATCTctgaAGTAAAAGTAACCCTGGAGAC3′

(forward) and

5′TTCAGACGTGTGCTCTTCCGATCTGACCAACTCAGCCAAAGTG3′

5′TTCAGACGTGTGCTCTTCCGATCTaGACCAACTCAGCCAAAGTG3′

5′TTCAGACGTGTGCTCTTCCGATCTgaGACCAACTCAGCCAAAGTG3′

5′TTCAGACGTGTGCTCTTCCGATCTtgaGACCAACTCAGCCAAAGTG3′

5′TTCAGACGTGTGCTCTTCCGATCTctgaGACCAACTCAGCCAAAGTG3′

(reverse), and for SHP2 (region around C459G)

5′CCCTACACGACGCTCTTCCGATCTGAGGTGCACCATAAGCAGGAG3′

5′CCCTACACGACGCTCTTCCGATCTaGAGGTGCACCATAAGCAGGAG3′

5′CCCTACACGACGCTCTTCCGATCTgaGAGGTGCACCATAAGCAGGAG3′

5′CCCTACACGACGCTCTTCCGATCTtgaGAGGTGCACCATAAGCAGGAG3′

5’CCCTACACGACGCTCTTCCGATCTctgaGAGGTGCACCATAAGCAGGAG3′

(forward) and

5′TTCAGACGTGTGCTCTTCCGATCTTCACAATGAACGTCCCTGTC3′

5′TTCAGACGTGTGCTCTTCCGATCTaTCACAATGAACGTCCCTGTC3′

5′TTCAGACGTGTGCTCTTCCGATCTgaTCACAATGAACGTCCCTGTC3′

5′TTCAGACGTGTGCTCTTCCGATCTtgaTCACAATGAACGTCCCTGTC3′

5′TTCAGACGTGTGCTCTTCCGATCTctgaTCACAATGAACGTCCCTGTC3′

(reverse).

The resulting amplicons were used for determination of cycle numbers for the second PCR with the TruSeq dual barcoding primers. The primary PCR product was amplified using NEBNext 2x High Fidelity PCR Master Mix (New England Biolabs), with 200nM primer concentration, and 0.1x SYBR Green I (SIGMA Aldrich; S9430) monitoring the amplification in a Roche LightCycler 480. The cycle number for the final library PCR was chosen to get a sufficient amount of product without reaching the plateau phase of the PCR. Then 2.5 μl of the primary PCR product was used for amplification with the NEBNext Master Mix and barcode primers (forward adaptor primers: 5′-AAT GAT ACG GCG ACC ACC GAG ATC TAC AC [8 bp barcode] ACA CTC TTT CCC TAC ACG ACG CTC TTC-3′; reverse adaptor primers: 5′-CAA GCA GAA GAC GGC ATA CGA GAT [8 bp barcode] GTG ACT GGA GTT CAG ACG TGT GCT CTT C-3′) but without SYBR Green, for the number of cycles determined from the qPCR. PCR products were purified using AMPure XP beads and concentration was determined on a Bioanalyzer DNA 1000 chip. Samples were sequenced using MiSeq v3 chemistry with 130 cycle single reads in a MiSeq sequencer (Illumina). Flanking reads were subtracted by mutant reads to calculate the reads corresponding to the endogenous expression level.

## Additional Information

**How to cite this article**: Beltran-Sastre, V. *et al.* Tuneable endogenous mammalian target complementation via multiplexed plasmid-based recombineering. *Sci. Rep.* **5**, 17432; doi: 10.1038/srep17432 (2015).

## Supplementary Material

Supplementary Information
