# Supplementary material for: Tuneable endogenous mammalian target complementation via multiplexed plasmid-based recombineering
Source: Sci Rep. 2015 Nov 27;5:17432. doi: 10.1038/srep17432 (PMC4661934; doi:10.1038/srep17432)
Supplement: Supplementary Information [file srep17432-s1.pdf]

Supplementary Information

Tuneable endogenous mammalian target complementation via multiplexed plasmid-based recombineering

Violeta Beltran-Sastre[1,2§], Hannah Benisty[1,2§], Julia Burnier[1,2], Imre Berger[3,4], Luis Serrano[1,2,5*], Christina Kiel[1,2*]

[1]EMBL/CRG Systems Biology Research Unit, Centre for Genomic Regulation (CRG), Dr. Aiguader 88, 08003 Barcelona, Spain

[2]Universitat Pompeu Fabra (UPF), 08003 Barcelona, Spain

[3]European Molecular Biology Laboratory, Grenoble Outstation, B.P. 181, Grenoble, France

[4]The School of Biochemistry, University of Bristol, Clifton BS8 1TD, United Kingdom

[5]Institució Catalana de Recerca i Estudis Avançats (ICREA), Pg. Lluís Companys 23, 08010 Barcelona, Spain

This file contains:
- Supplementary Figures 1 to 12
- Plasmid sequences

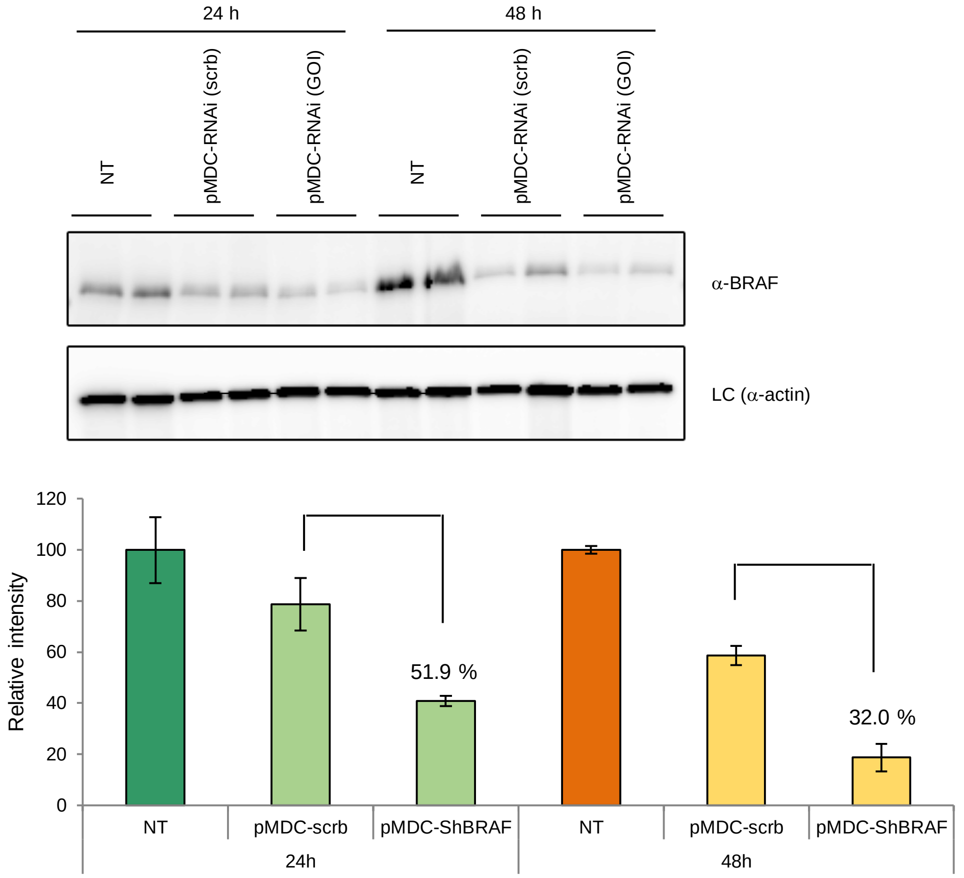

Supplementary Figure 1 | Biological replicates of BRAF downregulation HEK293 cells. shRNA-mediated downregulation of BRAF was analysed by Western blotting 24 h and 48 h after transfection with a Donor D1 producing specific shRNAs. Expression of BRAF was analyzed using ImageJ and normalized by expression of actin. Averages and standard deviations from two biological replicates are shown.

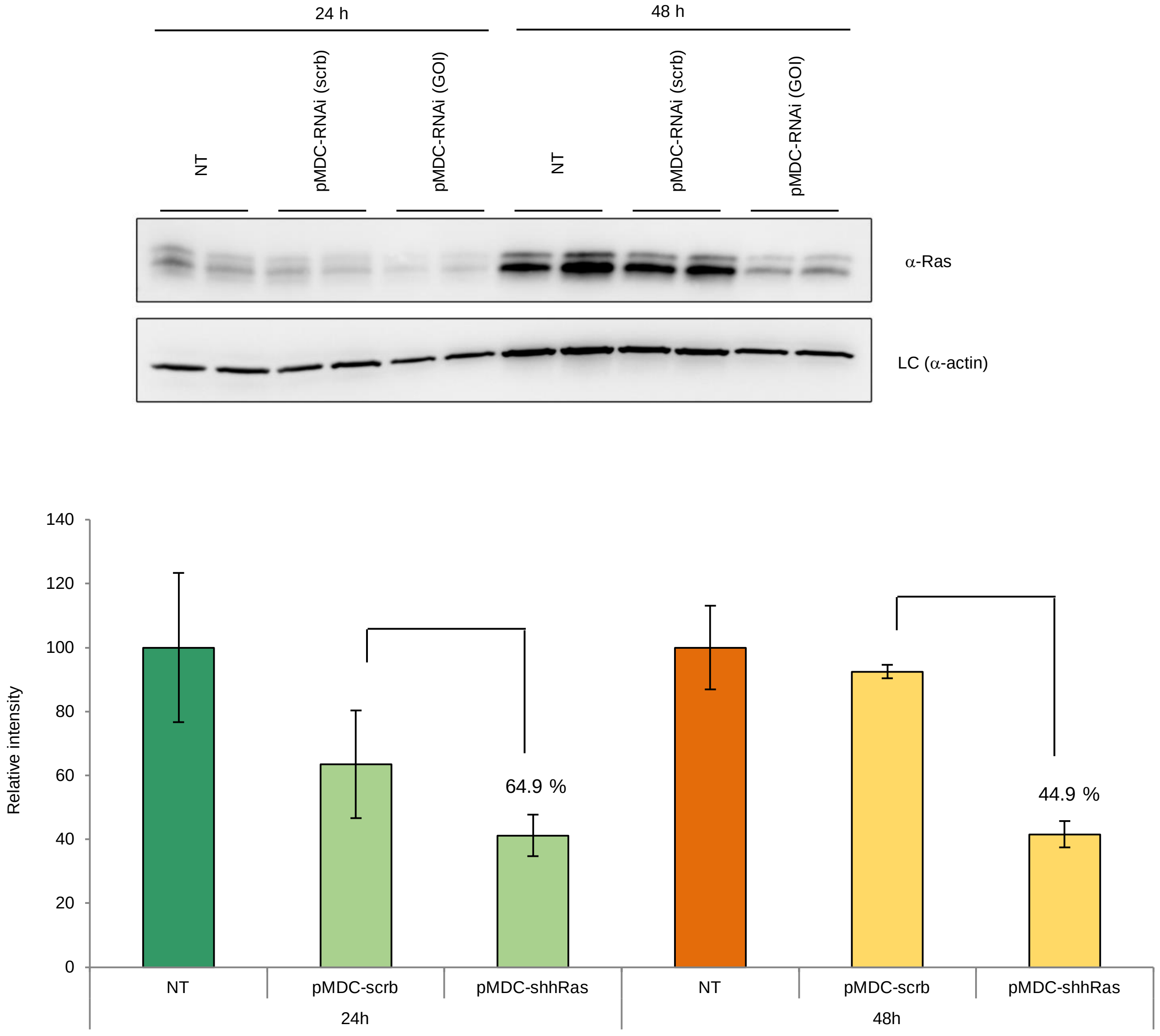

Supplementary Figure 2 | Biological replicates of RAS downregulation HEK293 cells. shRNA-mediated downregulation of RAS was analysed by Western blotting 24 h and 48 h after transfection with a Donor D1 producing specific shRNAs. Expression of Ras was analyzed using ImageJ and normalized by expression of actin. Averages and standard deviations from two biological replicates are shown.

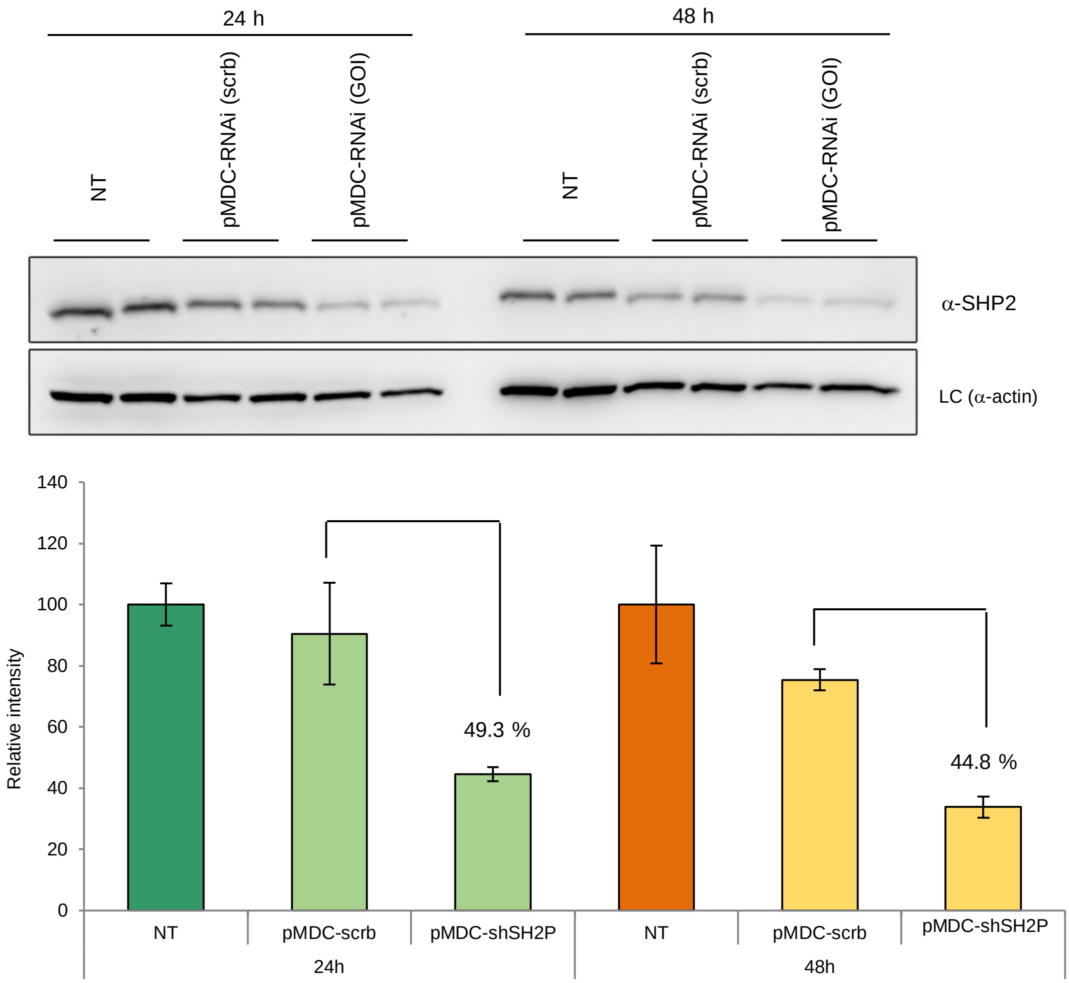

Supplementary Figure 3 | Biological replicates of SHP2 downregulation GH-HEK293 cells. shRNA-mediated downregulation of SHP2 was analysed by Western blotting 24 h and 48 h after transfection with a Donor D1 producing specific shRNAs. Expression of SHP2 was analyzed using ImageJ and normalized by expression of actin. Averages and standard deviations from two biological replicates are shown.

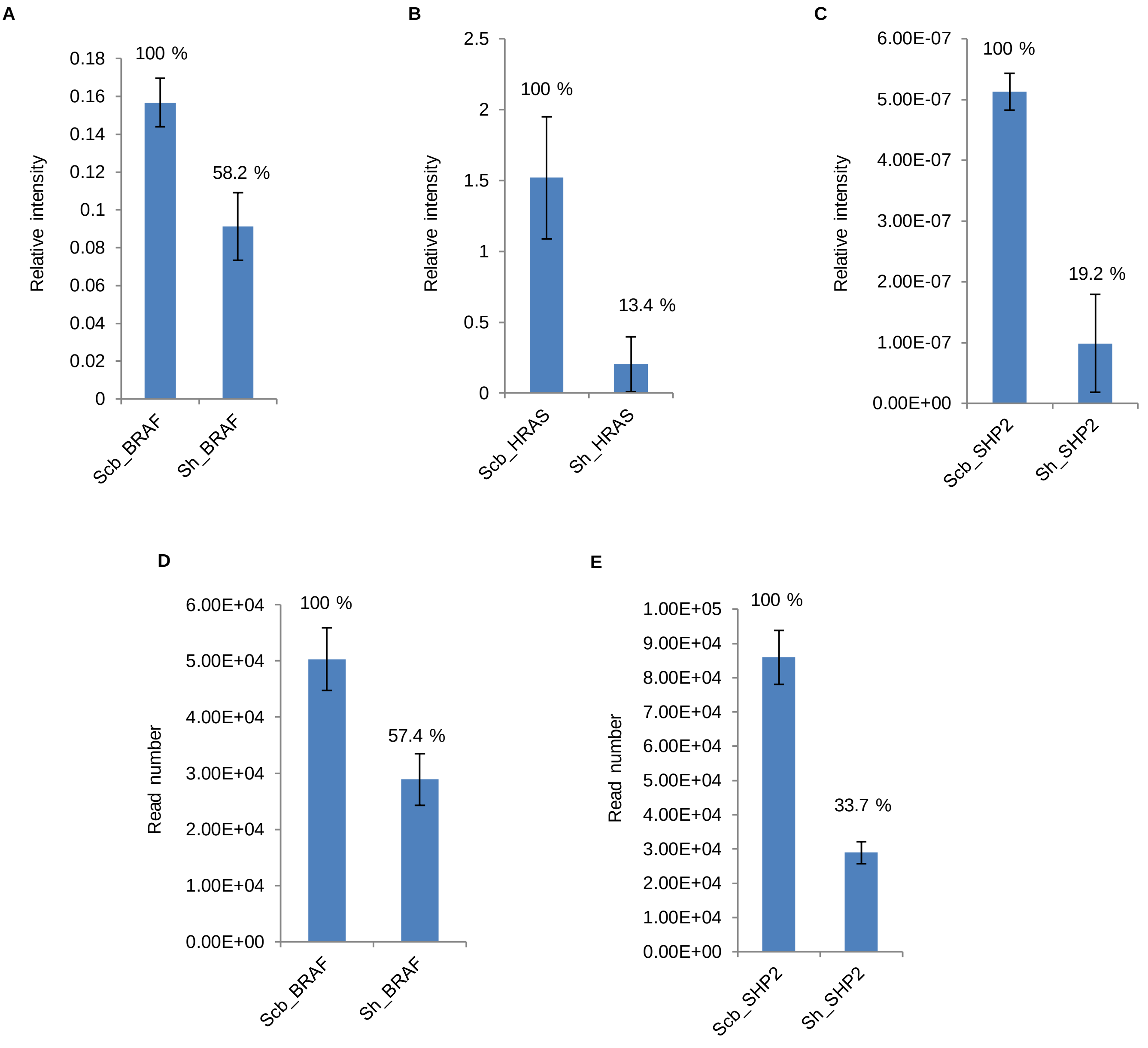

Supplementary Figure 4 | Downregulation of endogenous targets measured by qPCR and using calculated endogenous reads by Amplicon sequencing/MiSeq. (**A-C**). Results from qPCR using endogenous primer pairs. For BRAF (A), HRAS (B), and SHP2 (C). Transfection of sh (against target gene) and scb (scramble) were done using constructs of pTEMTAC at 0 ng/μl Dox concentration. 48 hours after transfection cells were analyzed and RNA was extracted. The endogenous levels were normalized by GADPH expression. Averages of three biological (for SHP2 two) and three technical replicates are shown. (**D-F**). Results of Amplicon PCR/MiSeq for BRAF V600E and SHP2 D61G only plotting endogenous reads with (sh) and without downregulation (Srb). Transfection of sh (against target gene) and scb (scramble) were done using constructs of pTEMTAC at 1 Dox concentration for SHP2 and 6 ng/μl Dox for BRAF. The endogenous reads are shown (flanking minus exogenous mutant reads).

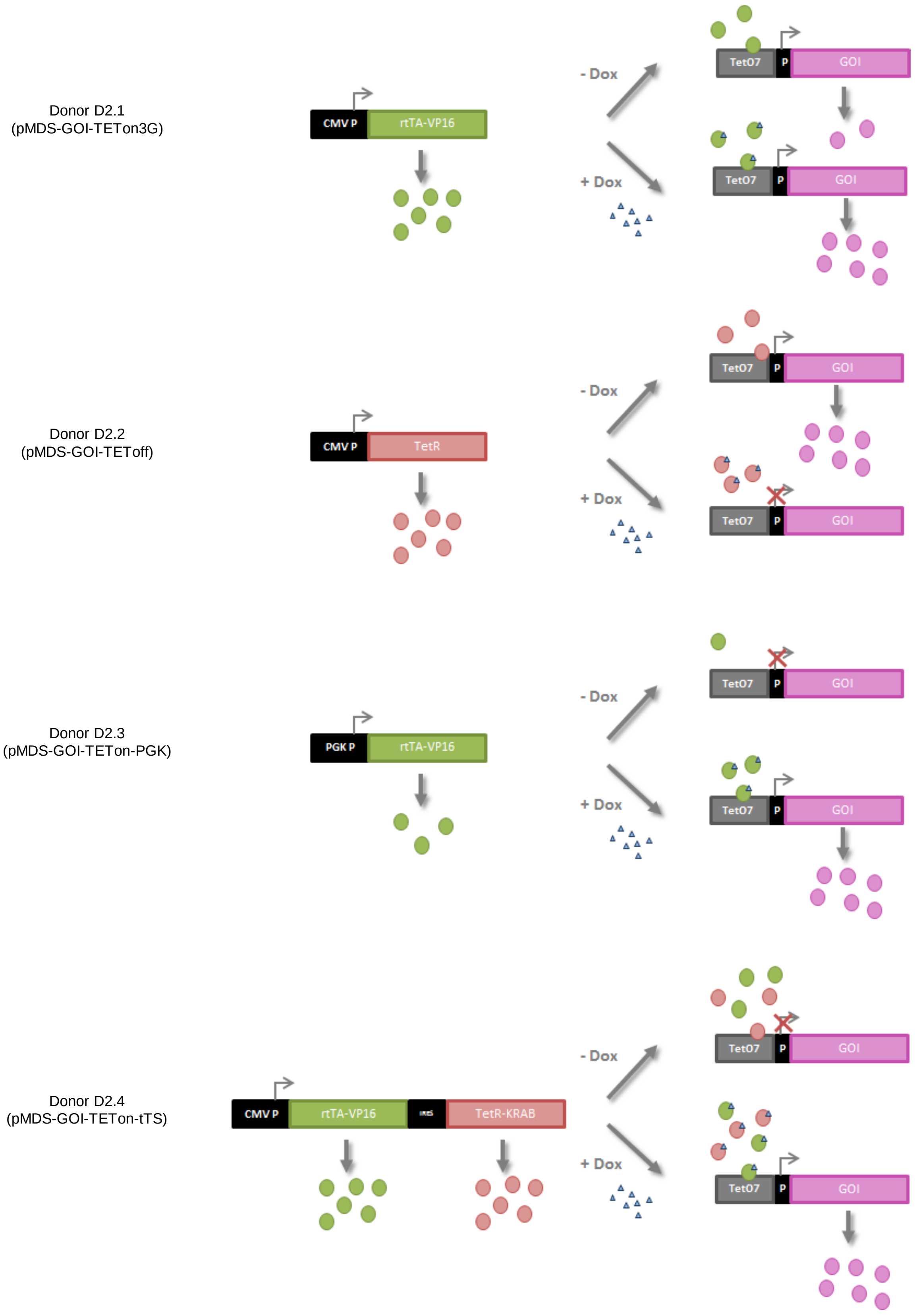

Supplementary Figure 5 | Schematic representation of gene expression regulation using Donor plasmids D2.1 to D2.4.

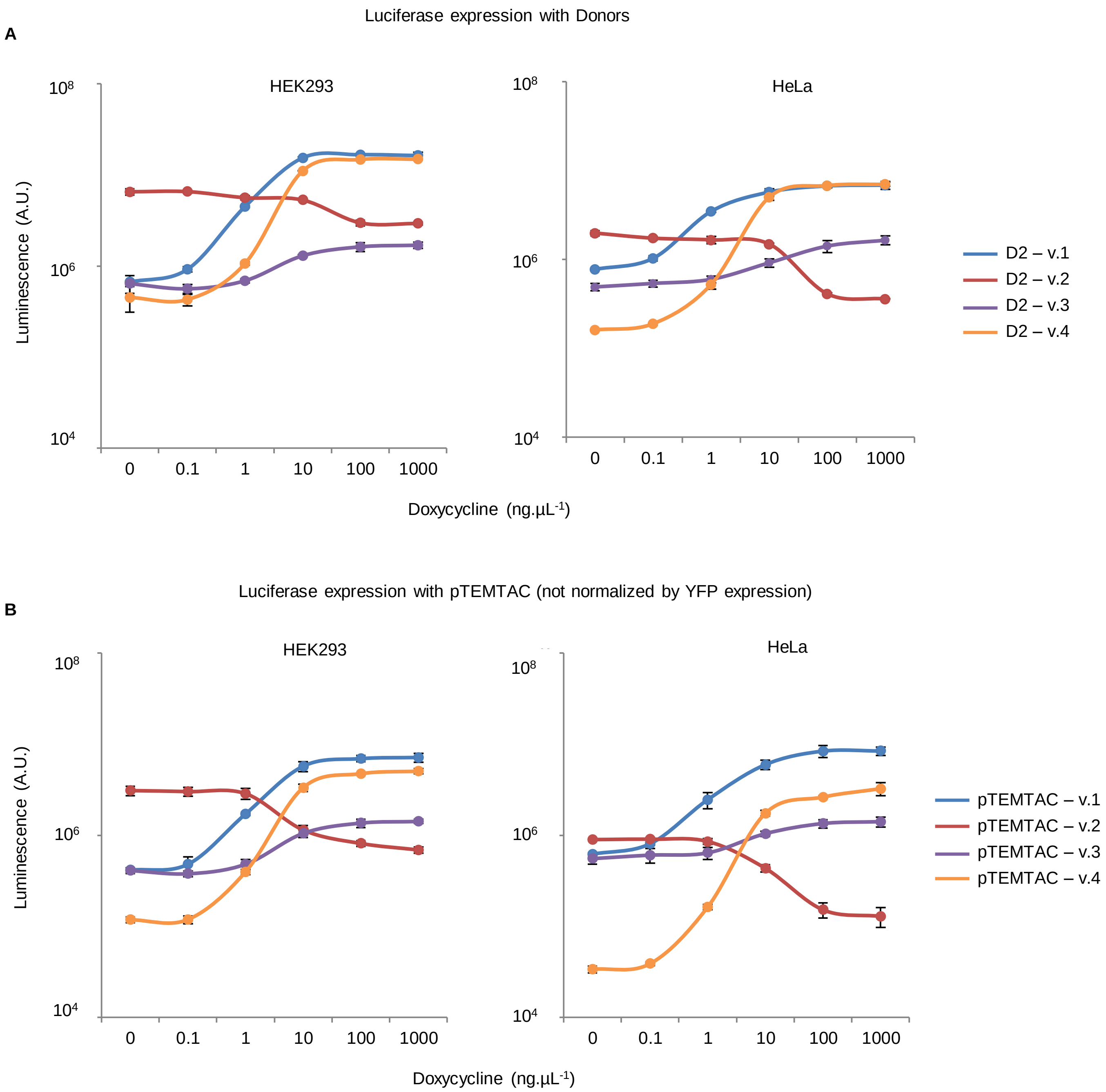

Supplementary Figure 6 | Luciferase expression with equimolar transfection of pTEMTAC or donor plasmids D2 – version 1 to 4 not normalized by YFP expression (for pTEMTAC experiments). (**A**) Luciferase assay results after transfection of HEK293 or HeLa with Donor D2 versions and docycycline induced expression of luciferase as GOI. (**B**). Similar as panel a, but with transfection of the full Cre-recombined plasmid (Donor D1 + Donore D2 + Acceptor A = pTEMTAC) not normalized by YFP expression. Averages and standard deviations from three biological replicates are shown.

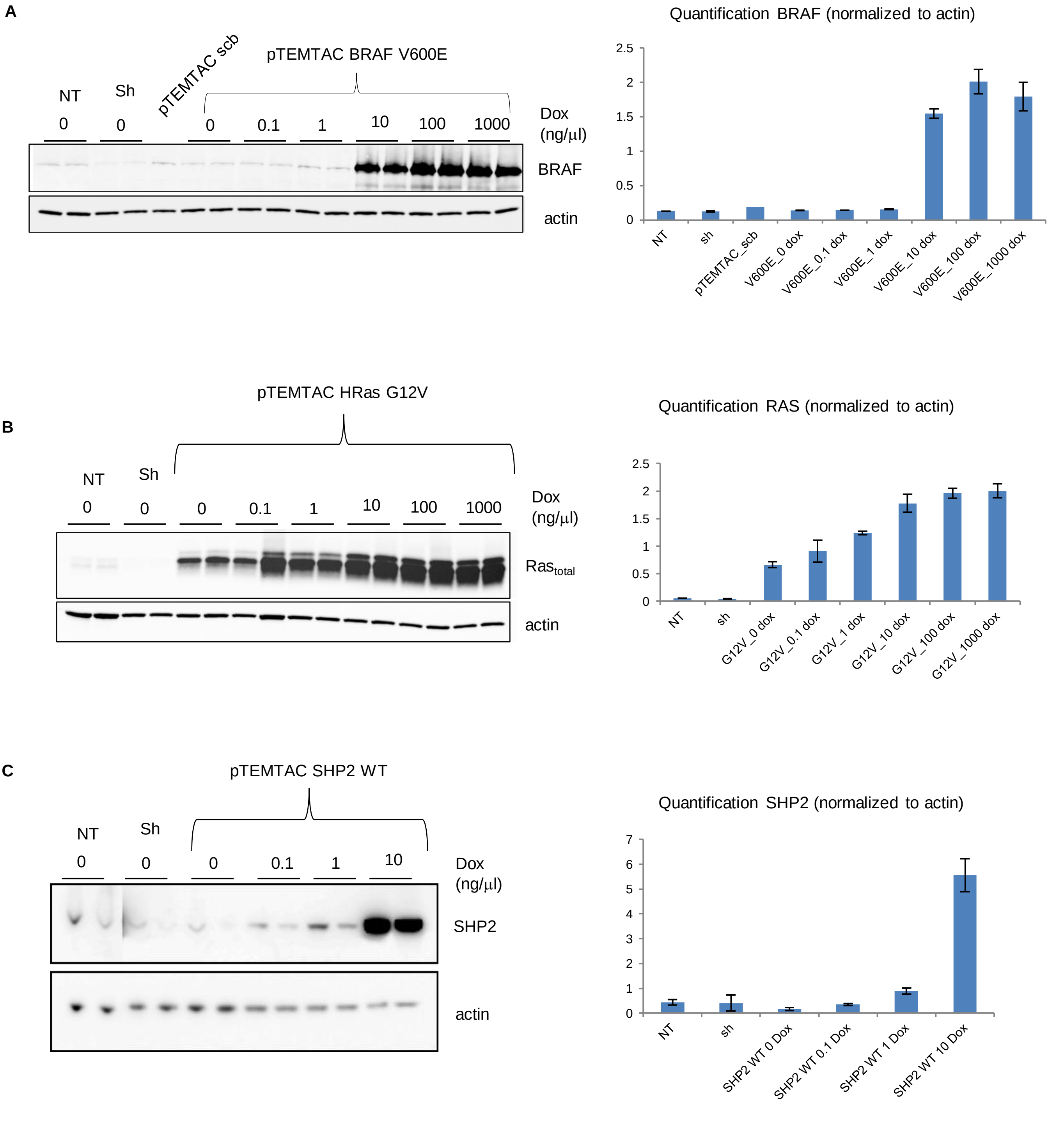

Supplementary Figure 7 | Expression of BRAF V600E, HRAS G12V and SHP2 WT using pTEMTAC with increasing Dox concentration and Western blot analysis. (**A**). Expression of pTEMTAC BRAF V600E using TETon-tTS in HEK293 cells. (**B**) Expression of pTEMTAC HRAS-G12V TETon3G in HEK293 cells. (**C**) Expression of pTEMTAC PTPN11 WT TETon3G in HEK293 cells.

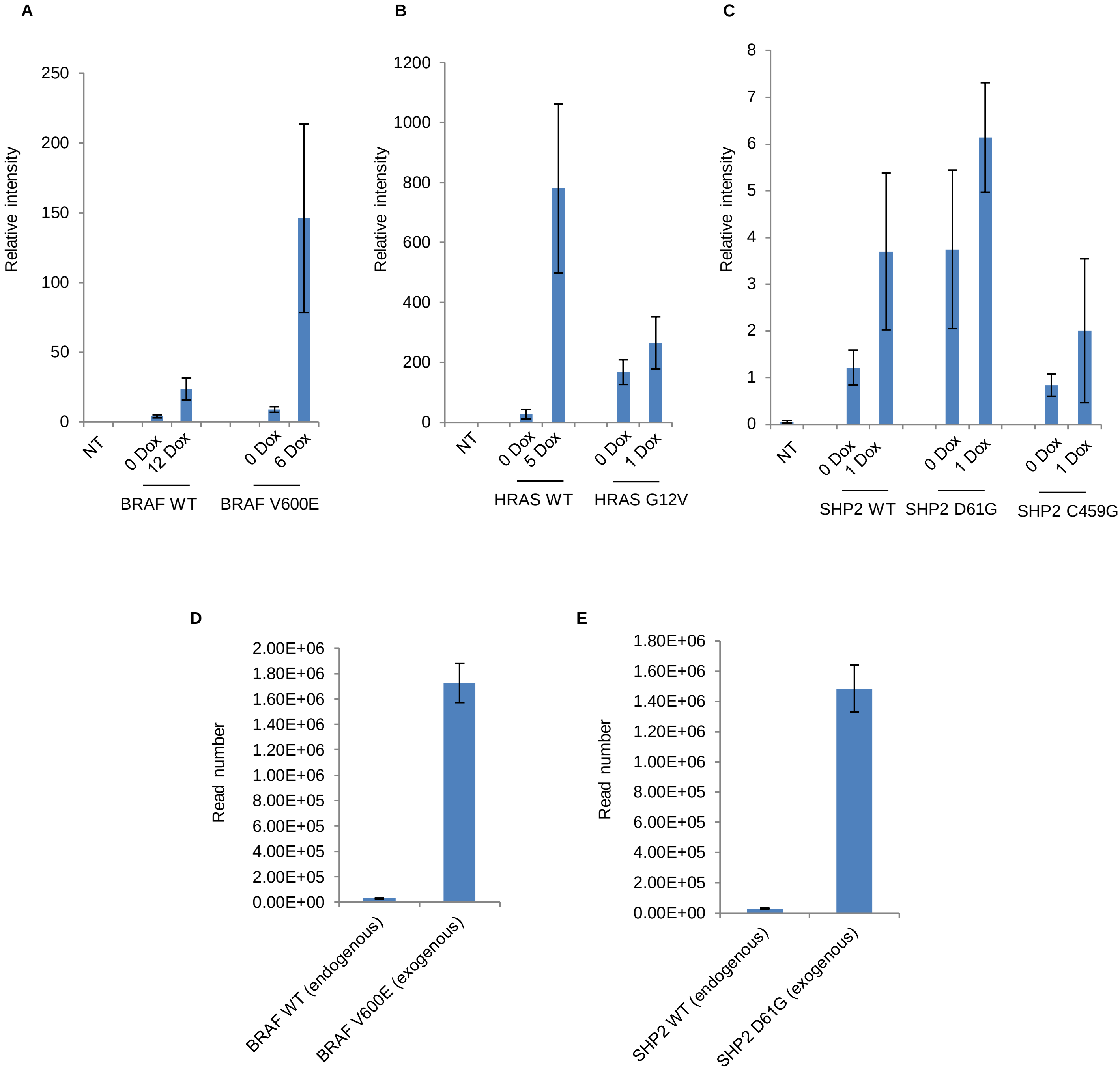

Supplementary Figure 8 | Exogenous expression of BRAF, HRAS and SHP2 using pTEMTAC measured by qPCR and Amplicon sequencing/MiSeq. (**A-C**) Results from qPCR. Expression of pTEMTAC Sh_BRAF WT and V600E using TETon-tTS in HEK293 cells without and with Dox concentration followed by RNA extraction and real time PCR using exogenous primers (panel A). Expression of pTEMTAC Sh_HRAS WT and G12V TETon3G in HEK293 cells (panel B). Expression of pTEMTAC Sh_PTPN11 WT, D61G, and C459G TETon3G in HEK293-GHR cells. (**D, E**). Results of Amplicon PCR/MiSeq for Sh_BRAF V600E and Sh_SHP2 D61G. Transfection were done using constructs of pTEMTAC at 1 ng/μl Dox concentration for Sh_SHP2 D61G and 6 ng/μl Dox for Sh_BRAF V600E. The mutant and endogenous (flanking minus exogenous mutant) reads are shown.

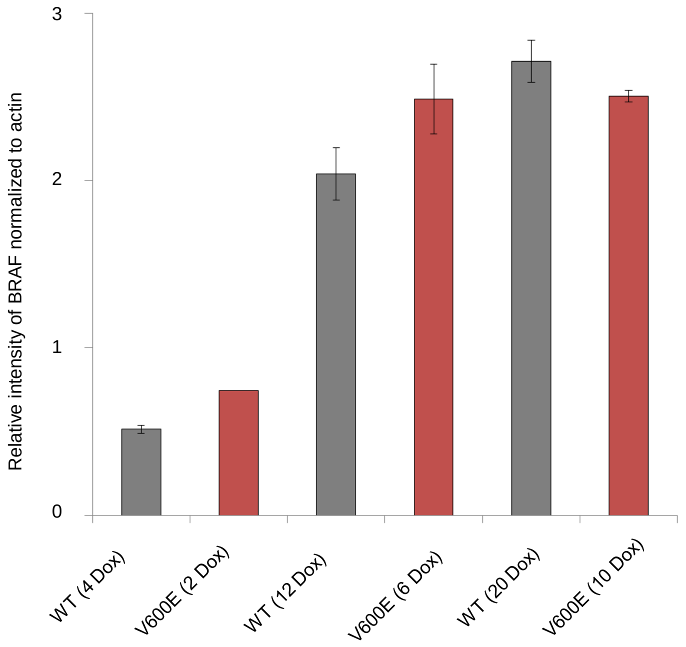

Supplementary Figure 9 | BRAF WT and V600E expression using different doxycycline concentrations. Quantification of the Expression of BRAF in HEK293 cells (see **Fig. 2a**) with a pTEMTAC plasmid (integration of Donor D2.4, pMDS-GOI-TETon-tTS). Averages and standard deviations from two biological replicates are shown.

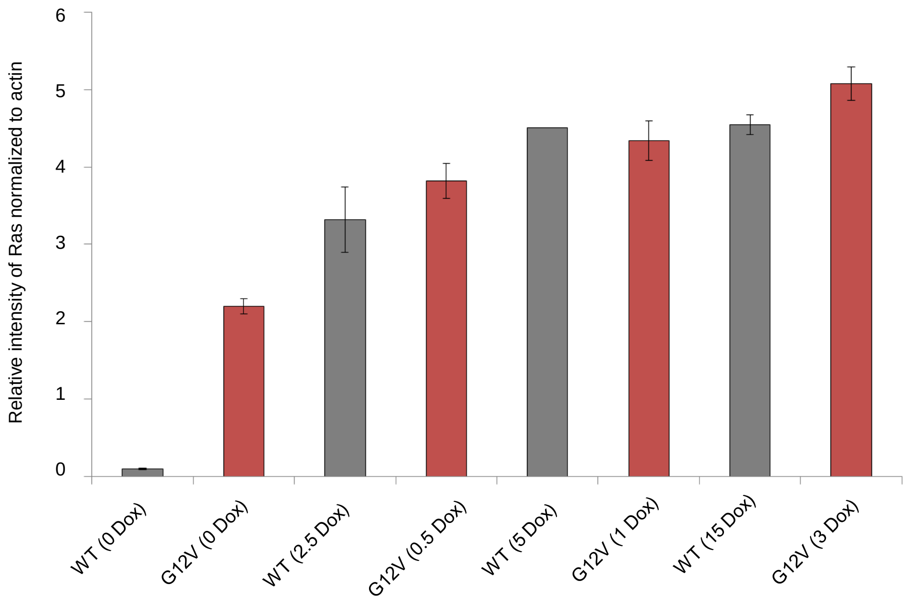

Supplementary Figure 10 | HRAS WT and G12V expression using different doxycycline concentrations. Quantification of the Expression of HRAS in HEK293 cells (see **Fig. 2b**) with a pTEMTAC plasmid (integration of Donor D2.1, pMDS-GOI-TETon). Averages and standard deviations from two biological replicates are shown.

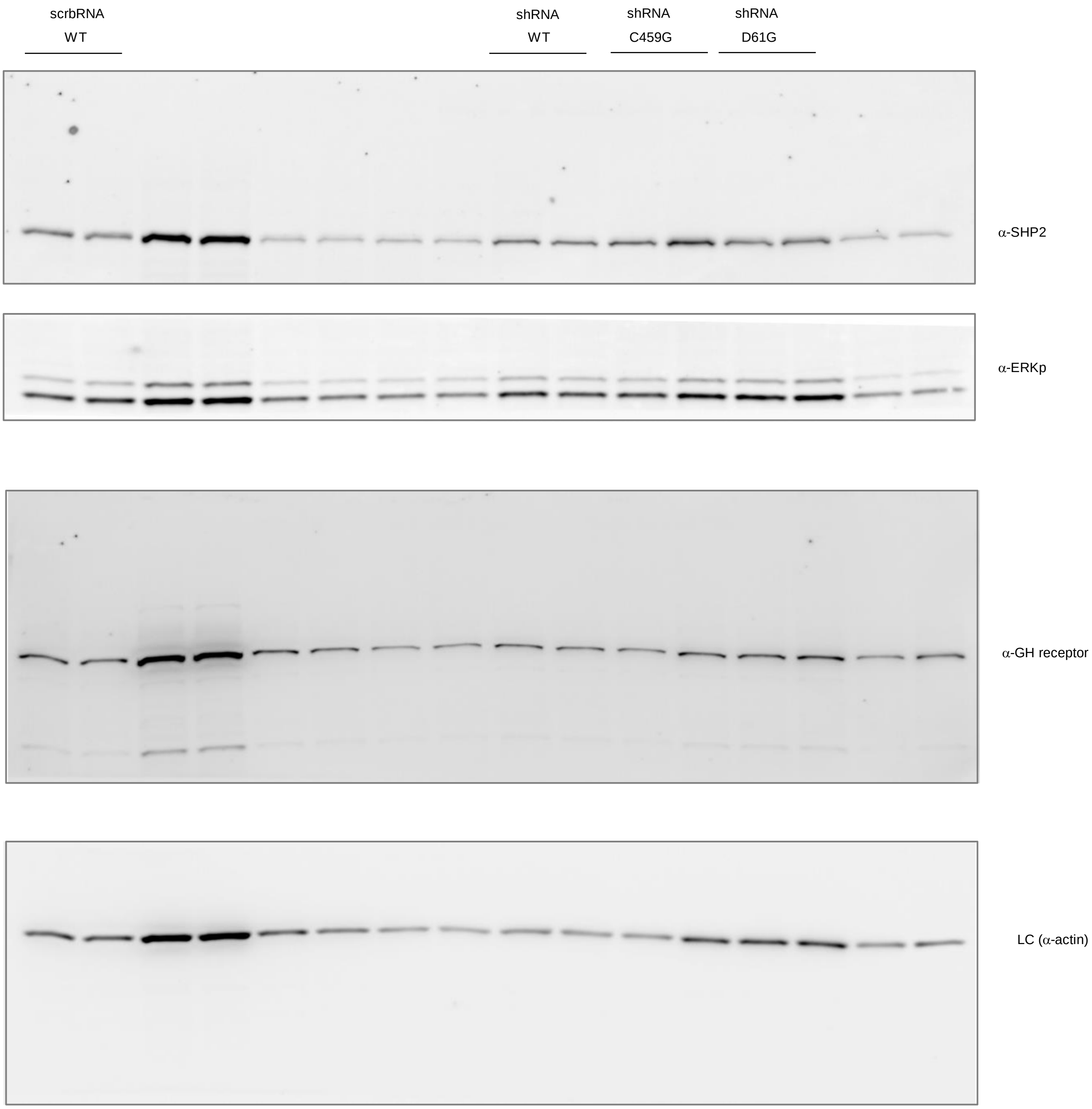

Supplementary Figure 11 | Non-cropped western blots for blots shown in main Figure 3C. See legend main figure.

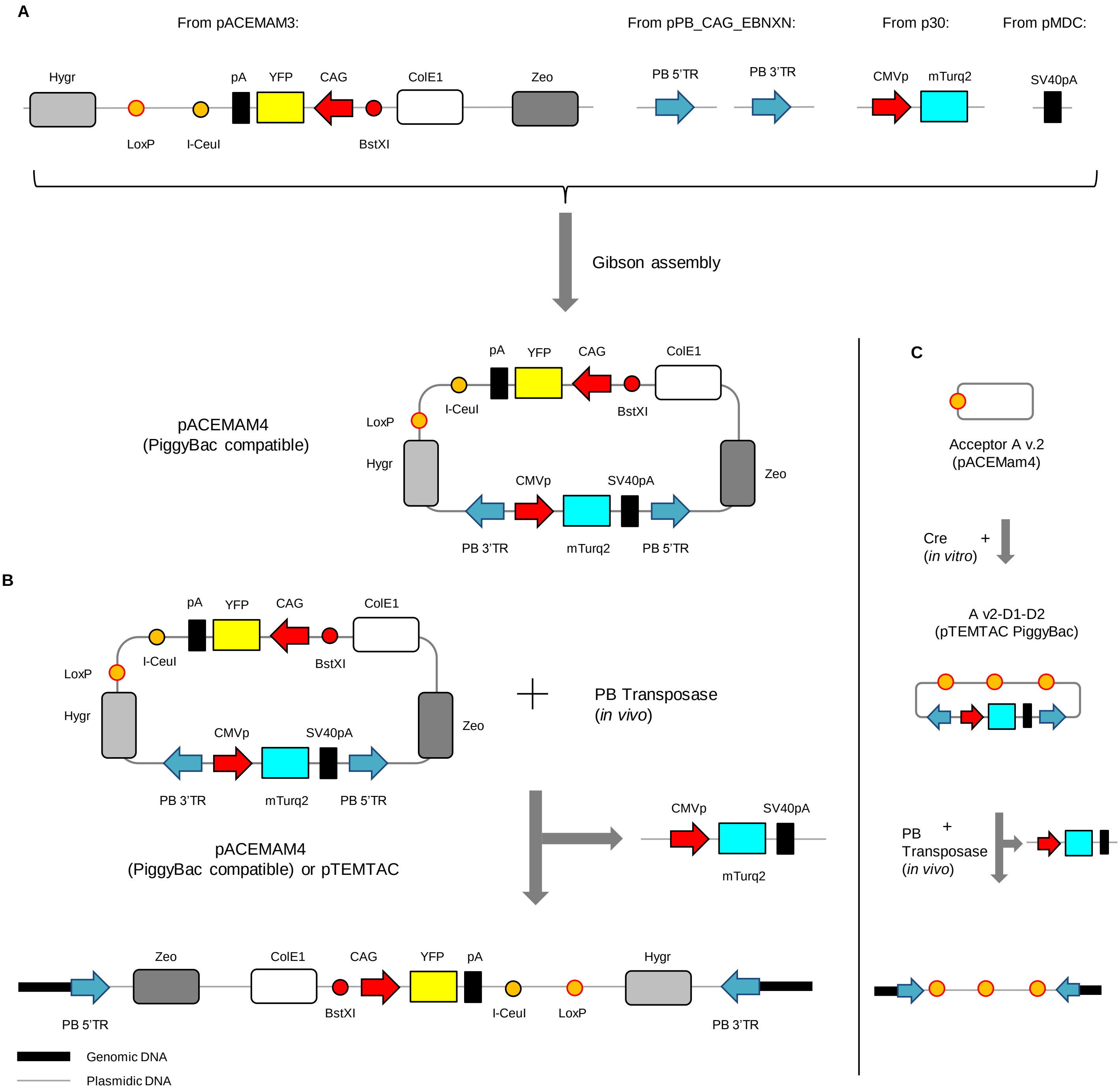

Supplementary Figure 12 | Assembly design for pACEMAM4 compatible with PiggyBac (Acceptor A v.2). (**A**) Schematic representation of PCR amplifications to generate the Acceptor pACEMAM4 using Gibson assembly. (**B, C**) Representation of integration of Acceptor A v.2 (panel A) or pTEMTAC PiggyBac (panel C) into genomic DNA in vivo using PB Transposase). All sequences are provided in the Supplementary Information. Abbreviations see main figure 1 and PB 5'TR; PiggyBac 5' terminal repeat; PB 3'TR; PiggyBac 3' terminal repeat; mTurq2; modified cyan fluorescent proteins; PB Transposase' PiggyBac Transposase.
References for pPB-CAG-EBNXN (Yusa et al, Nat Meth 6, 263-369, 2009), for p30 (Gruenberg et al, Nat Meth 6, 363-369, 2013).

Plasmid sequences:

<u>Content:</u>

Donor D1 (pMDC-RNAiDual):

DonorD2.1 (pMDS-GOI-TETon3G)

DonorD2.2 (pMDS-GOI-TEToff)

DonorD2.3 (pMDS-GOI-TETon-PGK)

DonorD2.4 (pMDS-GOI-TETon- tTS)

Acceptor A (pACEMam3)

AcceptorA v.2 (PiggyBac compatible) (pACEMAM4)

Donor D1 (pMDC-RNAiDual):

TGGCCCAGATCTATGTCGGGTGCGGAGAAAGAGGTAATGAAATGGCACCTAGGGCCCCCCCT
CGAGAAAAAGGGGATCCTGTGGTCTCATACAGAACTTATAAGATTCCCAAATCCAAAGACAT
TTCACGTTTATGGTGATTTCCCAGAACACATAGCGACATGCAAATATTGCAGGGCGCCACTC
CCCTGTCCCTCACAGCCATCTTCCTGCCAGGGCGCACGCGCGCTGGGTGTTCCCGCCTAGTG
ACACTGGGCCCGCGATTCCTTGGAGCGGGTTGATGACGTCAGCGTTCCGTACATCGCGAATC
GATACTAGGCCCAGTGGAAAGACGCGCAGGCAAAACGCACCACGTGACGGAGCGTGACCGCG
CGCCGAGCGCGCGCCAAGGTCGGGCAGGAAGAGGGCCTATTTCCCATGATTCCTTCATATTT
GCATATACGATACAAGGCTGTTAGAGAGATAATTAGAATTAATTTGACTGTAAACACAAAGA
TATTAGTACAAAATACGTGACGTAGAAAGTAATAATTTCTTGGGTAGTTTGCAGTTTTAAAA
TTATGTTTTAAAATGGACTATCATATGCTTACCGTAACTTGAAAGTATTTCGATTTCTTGGG
TTTATATATCTTGTGGAAAGGACGCGCTAGCGGATCCCATCTAGAACATCGCGAATCGATAC
TAGTAAAACCCATGTGCCTGGCAGATAACTTCGTATAATGTATGCTATACGAAGTTATGGTA
CCGCGGCCGCGTAGAGGATCTGTTGATCAGCAGTTCAACCTGTTGATAGTACGTACTAAGCT
CTCATGTTTCACGTACTAAGCTCTCATGTTTAACGTACTAAGCTCTCATGTTTAACGAACTA
AACCCTCATGGCTAACGTACTAAGCTCTCATGGCTAACGTACTAAGCTCTCATGTTTCACGT
ACTAAGCTCTCATGTTTGAACAATAAAATTAATATAAATCAGCAACTTAAATAGCCTCTAAG
GTTTTAAGTTTTATAAGAAAAAAAAGAATATATAAGGCTTTTAAAGCTTTTAAGGTTTAACG
GTTGTGGACAACAAGCCAGGGATGTAACGCACTGAGAAGCCCTTAGAGCCTCTCAAAGCAAT
TTTGAGTGACACAGGAACACTTAACGGCTGACATGGGAATTAGCTTCACGCTGCCGCAAGCA
CTCAGGGCGCAAGGGCTGCTAAAGGAAGCGGAACACGTAGAAAGCCAGTCCGCAGAAACGGT
GCTGACCCCGGATGAATGTCAGCTGGGAGGCAGAATAAATGATCATATCGTCAATTATTACC
TCCACGGGGAGAGCCTGAGCAAACTGGCCTCAGGCATTTGAGAAGCACACGGTCACACTGCT
TCCGGTAGTCAATAAACCGGTAAACCAGCAATAGACATAAGCGGCTATTTAACGACCCTGCC
CTGAACCGACGACCGGGTCGAATTTGCTTTCGAATTTCTGCCATTCATCCGCTTATTATCAC
TTATTCAGGCGTAGCAACCAGGCGTTTAAGGGCACCAATAACTGCCTTAAAAAAATTACGCC
CCGCCCTGCCACTCATCGCAGTACTGTTGTAATTCATTAAGCATTCTGCCGACATGGAAGCC
ATCACAAACGGCATGATGAACCTGAATCGCCAGCGGCATCAGCACCTTGTCGCCTTGCGTAT
AATATTTGCCCATGGTGAAAACGGGGGCGAAGAAGTTGTCCATATTGGCCACGTTTAAATCA
AAACTGGTGAAACTCACCCAGGGATTGGCTGAGACGAAAAACATATTCTCAATAAACCCTTT
AGGGAAATAGGCCAGGTTTTCACCGTAACACGCCACATCTTGCGAATATATGTGTAGAAACT
GCCGGAAATCGTCGTGGTATTCACTCCAGAGCGATGAAAACGTTTCAGTTTGCTCATGGAAA
ACGGTGTAACAAGGGTGAACACTATCCCATATCACCAGCTCACCGTCTTTCATTGCCATACG
GAATTCCGGATGAGCATTCATCAGGCGGGCAAGAATGTGAATAAAGGCCGGATAAAACTTGT
GCTTATTTTTCTTTACGGTCTTTAAAAAGGCCGTAATATCCAGCTGAACGGTCTGGTTATAG
GTACATTGAGCAACTGACTGAAATGCCTCAAAATGTTCTTTACGATGCCATTGGGATATATC
AACGGTGGTATATCCAGTGATTTTTTTCTCCATTTTAGCTTCCTTAGCTCCTGAAAATCTCG
ATAACTCAAAAAATACGCCCGGTAGTGATCTTATTTCATTATGGTGAAAGTTGGACCCTCTT
ACGTGCCGATCAACGTCTCATTTTCGCCAAAAGTTGGCCCAGATCAACGTCTCATTTTCGCC
AAAAGT

DonorD2.1 (pMDS-GOI-TETon3G):

CGATACTACGATACTAGTGCCCTTTCGTCTTCAAGAATTCCTCGAGTTTACTCCCTATCAGT
GATAGAGAACGTATGAAGAGTTTACTCCCTATCAGTGATAGAGAACGTATGCAGACTTTACT
CCCTATCAGTGATAGAGAACGTATAAGGAGTTTACTCCCTATCAGTGATAGAGAACGTATGA
CCAGTTTACTCCCTATCAGTGATAGAGAACGTATCTACAGTTTACTCCCTATCAGTGATAGA
GAACGTATATCCAGTTTACTCCCTATCAGTGATAGAGAACGTATAAGCTTTAGGCGTGTACG
GTGGGCGCCTATAAAAGCAGAGCTCGTTTAGTGAACCGTCAGATCGCCTGGAGCAATTCCAC
AACACTTTTGTCTTATACCAACTTTCCGTACCACTTCCTACCCTCGTAAACCCGGGATCTCG
AGCCATGGTGCTAGCAGCTGATGCATAGCATGCGGTACCTAATTCACTCCTCAGGTGCAGGC
TGCCTATCAGAAGGTGGTGGCTGGTGTGGCTAATGCCCTGGCTCACAAATACCACTGAGATC
TTTTTCCCTCTGCCAAAAATTATGGGGACATCATGAAGCCCCTTGAGCATCTGACTTCTGGC
TAATAAAGGAAATTTATTTTCATTGCAATAGTGTGTTGGAATTTTTTGTGTCTCTCACTCGG
AAGGACATATGGGAGGGCAAATCATTTAAAACATCAGAATGAGTATTTGGTTTAGAGTTTGG
CAACATATGCCATATGCTGGCTGCCATGAACAAAGGTGGCTATAAAGAGGTCATCAGTATAT
GAAACAGCCCCCTGCTGTCCATTCCTTATTCCATAGAAAAGCCTTGACTTGAGGTTAGATTT
TTTTTATATTTTGTTTTGTGTTATTTTTTTCTTTAACATCCCTAAAATTTTCCTTACATGTT
TTACTAGCCAGATTTTTCCTCCTCTCCTGACTACTCCCAGTCATAGCTGTCCCTCTTCTCTT
ATGAAGATCCCTCGACGTTTAAACCCATGTGCCTGGCAGATAACTTCGTATAATGTATGCTA
TACGAAGTTATGGTACGTACTAAGCTCTCATGTTTCACGTACTAAGCTCTCATGTTTAACGT
ACTAAGCTCTCATGTTTAACGAACTAAACCCTCATGGCTAACGTACTAAGCTCTCATGGCTA
ACGTACTAAGCTCTCATGTTTCACGTACTAAGCTCTCATGTTTGAACAATAAAATTAATATA
AATCAGCAACTTAAATAGCCTCTAAGGTTTTAAGTTTTATAAGAAAAAAAAGAATATATAAG
GCTTTTAAAGCTTTTAAGGTTTAACGGTTGTGGACAACAAGCCAGGGATGTAACGCACTGAG
AAGCCCTTAGAGCCTCTCAAAGCAATTTTGAGTGACACAGGAACACTTAACGGCTGACATAA
TTCAGCTTCACGCTGCCGCAAGCACTCAGGGCGCAAGGGCTGCTAAAGGAAGCGGAACACGT
AGAAAGCCAGTCCGCAGAAACGGTGCTGACCCCGGATGAATGTCAGCTGGGAGGCAGAATAA
ATGATCATATCGTCAATTATTACCTCCACGGGGAGAGCCTGAGCAAACTGGCCTCAGGCATT
TGAGAAGCACACGGTCACACTGCTTCCGGTAGTCAATAAACCGGTAAGTAGCGTATGCGCTC
ACGCAACTGGTCCAGAACCTTGACCGAACGCAGCGGTGGTAACGGCGCAGTGGCGGTTTTCA
TGGCTTGTTATGACTGTTTTTTTGGGGTACAGTCTATGCCTCGGGCATCCAAGCAGCAAGCG
CGTTACGCCGTGGGTCGATGTTTGATGTTATGGAGCAGCAACGATGTTACGCAGCAGGGCAG
TCGCCCTAAAACAAAGTTAAACATCATGAGGGAAGCGGTGATCGCCGAAGTATCGACTCAAC
TATCAGAGGTAGTTGGCGTCATCGAGCGCCATCTCGAACCGACGTTGCTGGCCGTACATTTG
TACGGCTCCGCAGTGGATGGCGGCCTGAAGCCACACAGTGATATTGATTTGCTGGTTACGGT
GACCGTAAGGCTTGATGAAACAACGCGGCGAGCTTTGATCAACGACCTTTTGGAAACTTCGG
CTTCCCCTGGAGAGAGCGAGATTCTCCGCGCTGTAGAAGTCACCATTGTTGTGCACGACGAC
ATCATTCCGTGGCGTTATCCAGCTAAGCGCGAACTGCAATTTGGAGAATGGCAGCGCAATGA
CATTCTTGCAGGTATCTTCGAGCCAGCCACGATCGACATTGATCTGGCTATCTTGCTGACAA
AAGCAAGAGAACATAGCGTTGCCTTGGTAGGTCCAGCGGCGGAGGAACTCTTTGATCCGGTT
CCTGAACAGGATCTATTTGAGGCGCTAAATGAAACCTTAACGCTATGGAACTCGCCGCCCGA
CTGGGCTGGCGATGAGCGAAATGTAGTGCTTACGTTGTCCCGCATTTGGTACAGCGCAGTAA
CCGGCAAAATCGCGCCGAAGGATGTCGCTGCCGACTGGGCAATGGAGCGCCTGCCGGCCCAG
TATCAGCCCGTCATACTTGAAGCTAGACAGGCTTATCTTGGACAAGAAGAAGATCGCTTGGC
CTCGCGCGCAGATCAGTTGGAAGAATTTGTCCACTACGTGAAAGGCGAGATCACCAAGGTAG
TCGGCAAATAATGTCTAACAATTCGTTCAAGCCGACGGATCTATGTCGGGTGCGGAGAAAGA

GGTAATGAAATGGCACCTAGGGGTTATGATAGTTATTGCTCAGCGGTGGCAGCAGCCAACTC
AGCTTCCTTTCGGGCCAAGCTTGGTCGAGCTGGATACTTCCCGTCCGCCAGGGGGACATGCC
GGCGATGCTGAAGGTCGCGCGCATTCCCGATGAAGAGGCCGGTTACCGCCTGTTGACCTGGT
GGGACGGGCAGGGCGCCGCCCGAGTCTTCGCCTCGGCGGCGGGCGCTCTGCTCATGGAGCGC
GCGTCCGGGGCCGGGGACCTTGCACAGATAGCGTGGTCCGGCCAGGACGACGAGGCTTGCAG
GATCATAATCAGCCATACCACATTTGTAGAGGTTTTACTTGCTTTAAAAAACCTCCCACACC
TCCCCCTGAACCTGAAACATAAAATGAATGCAATTGTTGTTGTTAACTTGTTTATTGCAGCT
TATAATGGTTACAAATAAAGCAATAGCATCACAAATTTCACAAATAAAGCATTTTTTTCACT
GCATTCTAGTTGTGGTTTGTCCAAACTCATCAATGTATCTTATCATGTCTGGATCCTTACTT
AGTTACCCGGGGAGCATGTCAAGGTCAAAATCGTCAAGAGCGTCAGCAGGCAGCATATCAAG
GTCAAAGTCGTCAAGGGCATCGGCTGGGAGCATGTCTAAGTCAAAATCGTCAAGGGCGTCGG
TCGGCCCGCCGCTTTCGCACTTTAGCTGTTTCTCCAGGCCACATATGATTAGTTCCAGGCCG
AAAAGGAAGGCAGGTTCGGCTCCCTGCCGGTCGAACAGCTCAATTGCTTGTTTCAGAAGTGG
GGGCATAGAATCGGTGGTAGGTGTCTCTCTTTCCTCTTTTGCTACTTGATGCTCCTGTTCCT
CCAATACGCAGCCCAGTGTAAAGTGGCCCACGGCGGACAGAGCGTACAGTGCGTTCTCCAGG
GAGAAGCCTTGCTGACACAGGAACGCGAGCTGATTTTCCAGGGTTTCGTACTGTTTCTCTGT
TGGGCGGGTGCCGAGATGCACTTTAGCCCCGTCGCGATGTGAGAGGAGAGCACAGCGGTATG
ACTTGGCGTTGTTCCGCAGAAAGTCTTGCCATGACTCGCCTTCCAGGGGGCAGGAGTGGGTA
TGATGCCTGTCCAGCATCTCGATTGGCAGGGCATCGAGCAGGGCCCGCTTGTTCTTCACGTG
CCAGTACAGGGTAGGCTGCTCAACTCCCAGCTTTTGAGCGAGTTTCCTTGTCGTCAGGCCTT
CGATACCGACTCCATTGAGTAATTCCAGAGCAGAGTTTATGACTTTGCTCTTGTCCAGTCTA
GACATGGTGAATTCCGCGCGCTTCGGACCGGGATCCACTAGCCAGCTTGGGTCTCCCTATAG
TGAGTCGTATTAATTTCGATAAGCCAGTAAGCAGTGGGTTCTCTAGTTAGCCAGAGAGCTCT
GCTTATATAGACCTCCCACCGTACACGCCTACCGCCCATTTGCGTCAATGGGGCGGAGTTGT
TACGACATTTTGGAAAGTCCCGTTGATTTTGGTGCCAAAACAAACTCCCATTGACGTCAATG
GGGTGGAGACTTGGAAATCCCCGTGAGTCAAACCGCTATCCACGCCCATTGATGTACTGCCA
AAACCGCATCACCATGGTAATAGCGATGACTAATACGTAGATGTACTGCCAAGTAGGAAAGT
CCCATAAGGTCATGTACTGGGCATAATGCCAGGCGGGCCATTTACCGTCATTGACGTCAATA
GGGGGCGTACTTGGCATATGATACACTTGATGTACTGCCAAGTGGGCAGTTTACCGTAAATA
CTCCACCCATTGACGTCAATGGAAAGTCCCTATTGGCGTTACTATGGGAACATACGTCATTA
TTGACGTCAATGGGCGGGGGTCGTTGGGCGGTCAGCCAGGCGGGCCATTTACCGTAAGTTAT
GTAACGCGGAACTCCATATATGGGCTATGAACTAATGACCCCGTAATTGATTACTATTAATA
ACTAGTCAATAATCAATGTCAACGCGTATATCTGGCCCGTACATCGCGAAT

DonorD2.2 (pMDS-GOI-TEToff):

CGATACTACGATACTAGTGCCCTTTCGTCTTCAAGAATTCCTGGCCCTTTCGTCTTCACTCG
AGTTTACTCCCTATCAGTGATAGAGAACGTATGTCGAGTTTACTCCCTATCAGTGATAGAGA
ACGATGTCGAGTTTACTCCCTATCAGTGATAGAGAACGTATGTCGAGTTTACTCCCTATCAG
TGATAGAGAACGTATGTCGAGTTTACTCCCTATCAGTGATAGAGAACGTATGTCGAGTTTAT
CCCTATCAGTGATAGAGAACGTATGTCGAGTTTACTCCCTATCAGTGATAGAGAACGTATGT
CGAGGTAGGCGTGTACGGTGGGAGGCCTATATAAGCAGAGCTCGTTTAGTGAACCGTCAGAT
CGCCTGGAGAATTCGAGCTCGGTACCCGGGGATCCTCTAGTCAGCTGACGCGTGCTAGCGCG
GCCGCATCGATAAGCTTGTCGACGATATCTCAGGTGCAGGCTGCCTATCAGAAGGTGGTGGC
TGGTGTGGCTAATGCCCTGGCTCACAAATACCACTGAGATCTTTTTCCCTCTGCCAAAAATT

ATGGGGACATCATGAAGCCCCTTGAGCATCTGACTTCTGGCTAATAAAGGAAATTTATTTTC
ATTGCAATAGTGTGTTGGAATTTTTTGTGTCTCTCACTCGGAAGGACATATGGGAGGGCAAA
TCATTTAAAACATCAGAATGAGTATTTGGTTTAGAGTTTGGCAACATATGCCATATGCTGGC
TGCCATGAACAAAGGTGGCTATAAAGAGGTCATCAGTATATGAAACAGCCCCCTGCTGTCCA
TTCCTTATTCCATAGAAAAGCCTTGACTTGAGGTTAGATTTTTTTTATATTTTGTTTTGTGT
TATTTTTTTCTTTAACATCCCTAAAATTTTCCTTACATGTTTTACTAGCCAGATTTTTCCTC
CTCTCCTGACTACTCCCAGTCATAGCTGTCCCTCTTCTCTTATGAAGATCCCTCGACGTTTA
AACCCATGTGCCTGGCAGATAACTTCGTATAATGTATGCTATACGAAGTTATGGTACGTACT
AAGCTCTCATGTTTCACGTACTAAGCTCTCATGTTTAACGTACTAAGCTCTCATGTTTAACG
AACTAAACCCTCATGGCTAACGTACTAAGCTCTCATGGCTAACGTACTAAGCTCTCATGTTT
CACGTACTAAGCTCTCATGTTTGAACAATAAAATTAATATAAATCAGCAACTTAAATAGCCT
CTAAGGTTTTAAGTTTTATAAGAAAAAAAAGAATATATAAGGCTTTTAAAGCTTTTAAGGTT
TAACGGTTGTGGACAACAAGCCAGGGATGTAACGCACTGAGAAGCCCTTAGAGCCTCTCAAA
GCAATTTTGAGTGACACAGGAACACTTAACGGCTGACATAATTCAGCTTCACGCTGCCGCAA
GCACTCAGGGCGCAAGGGCTGCTAAAGGAAGCGGAACACGTAGAAAGCCAGTCCGCAGAAAC
GGTGCTGACCCCGGATGAATGTCAGCTGGGAGGCAGAATAAATGATCATATCGTCAATTATT
ACCTCCACGGGGAGAGCCTGAGCAAACTGGCCTCAGGCATTTGAGAAGCACACGGTCACACT
GCTTCCGGTAGTCAATAAACCGGTAAGTAGCGTATGCGCTCACGCAACTGGTCCAGAACCTT
GACCGAACGCAGCGGTGGTAACGGCGCAGTGGCGGTTTTCATGGCTTGTTATGACTGTTTTT
TTGGGGTACAGTCTATGCCTCGGGCATCCAAGCAGCAAGCGCGTTACGCCGTGGGTCGATGT
TTGATGTTATGGAGCAGCAACGATGTTACGCAGCAGGGCAGTCGCCCTAAAACAAAGTTAAA
CATCATGAGGGAAGCGGTGATCGCCGAAGTATCGACTCAACTATCAGAGGTAGTTGGCGTCA
TCGAGCGCCATCTCGAACCGACGTTGCTGGCCGTACATTTGTACGGCTCCGCAGTGGATGGC
GGCCTGAAGCCACACAGTGATATTGATTTGCTGGTTACGGTGACCGTAAGGCTTGATGAAAC
AACGCGGCGAGCTTTGATCAACGACCTTTTGGAAACTTCGGCTTCCCCTGGAGAGAGCGAGA
TTCTCCGCGCTGTAGAAGTCACCATTGTTGTGCACGACGACATCATTCCGTGGCGTTATCCA
GCTAAGCGCGAACTGCAATTTGGAGAATGGCAGCGCAATGACATTCTTGCAGGTATCTTCGA
GCCAGCCACGATCGACATTGATCTGGCTATCTTGCTGACAAAAGCAAGAGAACATAGCGTTG
CCTTGGTAGGTCCAGCGGCGGAGGAACTCTTTGATCCGGTTCCTGAACAGGATCTATTTGAG
GCGCTAAATGAAACCTTAACGCTATGGAACTCGCCGCCCGACTGGGCTGGCGATGAGCGAAA
TGTAGTGCTTACGTTGTCCCGCATTTGGTACAGCGCAGTAACCGGCAAAATCGCGCCGAAGG
ATGTCGCTGCCGACTGGGCAATGGAGCGCCTGCCGGCCCAGTATCAGCCCGTCATACTTGAA
GCTAGACAGGCTTATCTTGGACAAGAAGAAGATCGCTTGGCCTCGCGCGCAGATCAGTTGGA
AGAATTTGTCCACTACGTGAAAGGCGAGATCACCAAGGTAGTCGGCAAATAATGTCTAACAA
TTCGTTCAAGCCGACGGATCTATGTCGGGTGCGGAGAAAGAGGTAATGAAATGGCACCTAGG
GGTTATGATAGTTATTGCTCAGCGGTGGCAGCAGCCAACTCAGCTTCCTTTCGGGCCAAGCT
TGGTCGAGCTGGATACTTCCCGTCCGCCAGGGGGACATGCCGGCGATGCTGAAGGTCGCGCG
CATTCCCGATGAAGAGGCCGGTTACCGCCTGTTGACCTGGTGGGACGGGCAGGGCGCCGCCC
GAGTCTTCGCCTCGGCGGCGGGCGCTCTGCTCATGGAGCGCGCGTCCGGGGCCGGGGACCTT
GCACAGATAGCGTGGTCCGGCCAGGACGACGAGGCTTGCAGGATCATAATCAGCCATACCAC
ATTTGTAGAGGTTTTACTTGCTTTAAAAAACCTCCCACACCTCCCCCTGAACCTGAAACATA
AAATGAATGCAATTGTTGTTGTTAACTTGTTTATTGCAGCTTATAATGGTTACAAATAAAGC
AATAGCATCACAAATTTCACAAATAAAGCATTTTTTTCACTGCATTCTAGTTGTGGTTTGTC
CAAACTCATCAATGTATCTTATCATGTCTGGATCCTTACTTAGTTACCCGGGGAGCATGTCA

AGGTCAAAATCGTCAAGAGCGTCAGCAGGCAGCATATCAAGGTCAAAGTCGTCAAGGGCATC
GGCTGGGAGCATGTCTAAGTCAAAATCGTCAAGGGCGTCGGCCGGCCCGCCGCTTTCGCACT
TTAGCTGTTTCTCCAGGCCACATATGATTAGTTCCAGGCCGAAAAGGAAGGCAGGTTCGGCT
CCCTGATGGTCGAACAGCTCAATTGCTTGTCTCAGAAGTGGGGGCATAGAATCGGTGGTAGG
TGTCTCTCTTTCCTCTTTTGCTACTTGATGCTCCTGATCCTCCAATACGCAGCCCAGTGTAA
AGTGGCCCACGGCGGACAGAGCGTACAGTGCGTTCTCCAGGGAGAAGCCTTGCTGACACAGG
AACGCGAGCTGATTTTCCAGGGTTTCGTACTGTTTCTCTGTTGGGCGGGTGCCGAGATGCAC
TTTAGCCCCGTCGCGATGTGAGAGGAGAGCACAGCGGAATGACTTGGCGTTGTTCCGCAGAA
AGTCTTGCCATGACTCGCCTTCCAGGGGGCAGAAGTGGGTATGATGCCTGTCCAGCATCTCG
ATTGCCAGGGCATCGAGCAGGGCCCGCTTGTTCTTCACGTGCCAGTACAGGGTAGGCTGCTC
AACTCCCAGCTTTTGAGCGAGTTTCCTTGTCGTCAGGCCTTCGATACCGACTTCATTGAGTA
ATTCCAGAGCAGAGTTTATGACTTTGCTCTTGTCCAGTCTAGACATGGTGAATTCGGGGCCG
CGGAGGCTGGATCGGTCCCGGTGTCTTCTATGGAGGTCAAAACAGCGTGGATGGCGTCTCCA
GGCGATCTGACGGTTCACTAAACGAGCTCTGCTTATATAGACCTCCCACCGTACACGCCTAC
CGCCCATTTGCGTCAATGGGGCGGAGTTGTTACGACATTTTGGAAAGTCCCGTTGATTTTGG
TGCCAAAACAAACTCCCATTGACGTCAATGGGGTGGAGACTTGGAAATCCCCGTGAGTCAAA
CCGCTATCCACGCCCATTGATGTACTGCCAAAACCGCATCACCATGGTAATAGCGATGACTA
ATACGTAGATGTACTGCCAAGTAGGAAAGTCCCATAAGGTCATGTACTGGGCATAATGCCAG
GCGGGCCATTTACCGTCATTGACGTCAATAGGGGGCGTACTTGGCATATGATACACTTGATG
TACTGCCAAGTGGGCAGTTTACCGTAAATACTCCACCCATTGACGTCAATGGAAAGTCCCTA
TTGGCGTTACTATGGGAACATACGTCATTATTGACGTCAATGGGCGGGGGTCGTTGGGCGGT
CAGCCAGGCGGGCCATTTACCGTAAGTTATGTAACGCGGAACTCCATATATGGGCTATGAAC
TAATGACCCCGTAATTGATTACTATTAATAACTAGTCAATAATCAATGTCAACGCGTATATC
TGGCCCGTACATCGCGAAT

DonorD2.3 (pMDS-GOI-TETon-PGK):

CGATACTACGATACTAGTGCCCTTTCGTCTTCAAGAATTCCTCGAGTTTACTCCCTATCAGT
GATAGAGAACGTATGAAGAGTTTACTCCCTATCAGTGATAGAGAACGTATGCAGACTTTACT
CCCTATCAGTGATAGAGAACGTATAAGGAGTTTACTCCCTATCAGTGATAGAGAACGTATGA
CCAGTTTACTCCCTATCAGTGATAGAGAACGTATCTACAGTTTACTCCCTATCAGTGATAGA
GAACGTATATCCAGTTTACTCCCTATCAGTGATAGAGAACGTATAAGCTTTTCTACCGGGTA
GGGGAGGCGCTTTTCCCAAGGCAGTCTGGAGCATGCGCTTTAGCAGCCCCGCTGGGCACTTG
GCGCTACACAAGTGGCCTCTGGCCTCGCACACATTCCACATCCACCGGTAGGCGCCAACCGG
CTCCGTTCTTTGGTGGCCCCTTCGCGCCACCTTCTACTCCTCCCCTAGTCAGGAAGTTCCCC
CCCGCCCCGCAGCTCGCGTCGTGCAGGACGTGACAAATGGAAGTAGCACGTCTCACTAGTCT
CGTGCAGATGGACAGCACCGCTGAGCAATGGAAGCGGGTAGGCCTTTGGGGCAGCGGCCAAT
AGCAGCTTTGCTCCTTCGCTTTCTGGGCTCAGAGGCTGGGAAGGGGTGGGTCCGGGGGCGGG
CTCAGGGGCGGGCTCAGGGGCGGGGCGGGCGCCCGAAGGTCCTCCGGAGGCCCGGCATTCTG
CACGCTTCAAAAGCGCACGTCTGCCGCGCTGTTCTCCTCTTCCTCATCTCCGGGCCTTTCGA
CCTCCCGGGATCTCGAGCCATGGTGCTAGCAGCTGATGCATAGCATGCGGTACCTAATTCAC
TCCTCAGGTGCAGGCTGCCTATCAGAAGGTGGTGGCTGGTGTGGCTAATGCCCTGGCTCACA
AATACCACTGAGATCTTTTTCCCTCTGCCAAAAATTATGGGGACATCATGAAGCCCCTTGAG
CATCTGACTTCTGGCTAATAAAGGAAATTTATTTTCATTGCAATAGTGTGTTGGAATTTTTT
GTGTCTCTCACTCGGAAGGACATATGGGAGGGCAAATCATTTAAAACATCAGAATGAGTATT

TGGTTTAGAGTTTGGCAACATATGCCATATGCTGGCTGCCATGAACAAAGGTGGCTATAAAG
AGGTCATCAGTATATGAAACAGCCCCCTGCTGTCCATTCCTTATTCCATAGAAAAGCCTTGA
CTTGAGGTTAGATTTTTTTTATATTTTGTTTTGTGTTATTTTTTTCTTTAACATCCCTAAAA
TTTTCCTTACATGTTTTACTAGCCAGATTTTTCCTCCTCTCCTGACTACTCCCAGTCATAGC
TGTCCCTCTTCTCTTATGAAGATCCCTCGACGTTTAAACCCATGTGCCTGGCAGATAACTTC
GTATAATGTATGCTATACGAAGTTATGGTACGTACTAAGCTCTCATGTTTCACGTACTAAGC
TCTCATGTTTAACGTACTAAGCTCTCATGTTTAACGAACTAAACCCTCATGGCTAACGTACT
AAGCTCTCATGGCTAACGTACTAAGCTCTCATGTTTCACGTACTAAGCTCTCATGTTTGAAC
AATAAAATTAATATAAATCAGCAACTTAAATAGCCTCTAAGGTTTTAAGTTTTATAAGAAAA
AAAAGAATATATAAGGCTTTTAAAGCTTTTAAGGTTTAACGGTTGTGGACAACAAGCCAGGG
ATGTAACGCACTGAGAAGCCCTTAGAGCCTCTCAAAGCAATTTTGAGTGACACAGGAACACT
TAACGGCTGACATAATTCAGCTTCACGCTGCCGCAAGCACTCAGGGCGCAAGGGCTGCTAAA
GGAAGCGGAACACGTAGAAAGCCAGTCCGCAGAAACGGTGCTGACCCCGGATGAATGTCAGC
TGGGAGGCAGAATAAATGATCATATCGTCAATTATTACCTCCACGGGGAGAGCCTGAGCAAA
CTGGCCTCAGGCATTTGAGAAGCACACGGTCACACTGCTTCCGGTAGTCAATAAACCGGTAA
GTAGCGTATGCGCTCACGCAACTGGTCCAGAACCTTGACCGAACGCAGCGGTGGTAACGGCG
CAGTGGCGGTTTTCATGGCTTGTTATGACTGTTTTTTTGGGGTACAGTCTATGCCTCGGGCA
TCCAAGCAGCAAGCGCGTTACGCCGTGGGTCGATGTTTGATGTTATGGAGCAGCAACGATGT
TACGCAGCAGGGCAGTCGCCCTAAAACAAAGTTAAACATCATGAGGGAAGCGGTGATCGCCG
AAGTATCGACTCAACTATCAGAGGTAGTTGGCGTCATCGAGCGCCATCTCGAACCGACGTTG
CTGGCCGTACATTTGTACGGCTCCGCAGTGGATGGCGGCCTGAAGCCACACAGTGATATTGA
TTTGCTGGTTACGGTGACCGTAAGGCTTGATGAAACAACGCGGCGAGCTTTGATCAACGACC
TTTTGGAAACTTCGGCTTCCCCTGGAGAGAGCGAGATTCTCCGCGCTGTAGAAGTCACCATT
GTTGTGCACGACGACATCATTCCGTGGCGTTATCCAGCTAAGCGCGAACTGCAATTTGGAGA
ATGGCAGCGCAATGACATTCTTGCAGGTATCTTCGAGCCAGCCACGATCGACATTGATCTGG
CTATCTTGCTGACAAAAGCAAGAGAACATAGCGTTGCCTTGGTAGGTCCAGCGGCGGAGGAA
CTCTTTGATCCGGTTCCTGAACAGGATCTATTTGAGGCGCTAAATGAAACCTTAACGCTATG
GAACTCGCCGCCCGACTGGGCTGGCGATGAGCGAAATGTAGTGCTTACGTTGTCCCGCATTT
GGTACAGCGCAGTAACCGGCAAAATCGCGCCGAAGGATGTCGCTGCCGACTGGGCAATGGAG
CGCCTGCCGGCCCAGTATCAGCCCGTCATACTTGAAGCTAGACAGGCTTATCTTGGACAAGA
AGAAGATCGCTTGGCCTCGCGCGCAGATCAGTTGGAAGAATTTGTCCACTACGTGAAAGGCG
AGATCACCAAGGTAGTCGGCAAATAATGTCTAACAATTCGTTCAAGCCGACGGATCTATGTC
GGGTGCGGAGAAAGAGGTAATGAAATGGCACCTAGGGGTTATGATAGTTATTGCTCAGCGGT
GGCAGCAGCCAACTCAGCTTCCTTTCGGGCCAAGCTTGGTCGAGCTGGATACTTCCCGTCCG
CCAGGGGGACATGCCGGCGATGCTGAAGGTCGCGCGCATTCCCGATGAAGAGGCCGGTTACC
GCCTGTTGACCTGGTGGGACGGGCAGGGCGCCGCCCGAGTCTTCGCCTCGGCGGCGGGCGCT
CTGCTCATGGAGCGCGCGTCCGGGGCCGGGGACCTTGCACAGATAGCGTGGTCCGGCCAGGA
CGACGAGGCTTGCAGGATCATAATCAGCCATACCACATTTGTAGAGGTTTTACTTGCTTTAA
AAAACCTCCCACACCTCCCCCTGAACCTGAAACATAAAATGAATGCAATTGTTGTTGTTAAC
TTGTTTATTGCAGCTTATAATGGTTACAAATAAAGCAATAGCATCACAAATTTCACAAATAA
AGCATTTTTTTCACTGCATTCTAGTTGTGGTTTGTCCAAACTCATCAATGTATCTTATCATG
TCTGGATCCTTACTTAGTTACCCGGGGAGCATGTCAAGGTCAAAATCGTCAAGAGCGTCAGC
AGGCAGCATATCAAGGTCAAAGTCGTCAAGGGCATCGGCTGGGAGCATGTCTAAGTCAAAAT
CGTCAAGGGCGTCGGTCGGCCCGCCGCTTTCGCACTTTAGCTGTTTCTCCAGGCCACATATG

ATTAGTTCCAGGCCGAAAAGGAAGGCAGGTTCGGCTCCCTGCCGGTCGAACAGCTCAATTGC
TTGTTTCAGAAGTGGGGGCATAGAATCGGTGGTAGGTGTCTCTCTTTCCTCTTTTGCTACTT
GATGCTCCTGTTCCTCCAATACGCAGCCCAGTGTAAAGTGGCCCACGGCGGACAGAGCGTAC
AGTGCGTTCTCCAGGGAGAAGCCTTGCTGACACAGGAACGCGAGCTGATTTTCCAGGGTTTC
GTACTGTTTCTCTGTTGGGCGGGTGCCGAGATGCACTTTAGCCCCGTCGCGATGTGAGAGGA
GAGCACAGCGGTATGACTTGGCGTTGTTCCGCAGAAAGTCTTGCCATGACTCGCCTTCCAGG
GGGCAGGAGTGGGTATGATGCCTGTCCAGCATCTCGATTGGCAGGGCATCGAGCAGGGCCCG
CTTGTTCTTCACGTGCCAGTACAGGGTAGGCTGCTCAACTCCCAGCTTTTGAGCGAGTTTCC
TTGTCGTCAGGCCTTCGATACCGACTCCATTGAGTAATTCCAGAGCAGAGTTTATGACTTTG
CTCTTGTCCAGTCTAGACATGGTGAATTCCGCGCGCTTCGGACCGGGATCCACTAGCCAGCT
TGGGTCTCCCTATAGTGAGTCGTATTAATTTCGATAAGCCAGTAAGCAGTGGGTTCTCTAGT
TAGCCAGAGAGCTCTGCTTATATAGACCTCCCACCGTACACGCCTACCGCCCATTTGCGTCA
ATGGGGCGGAGTTGTTACGACATTTTGGAAAGTCCCGTTGATTTTGGTGCCAAAACAAACTC
CCATTGACGTCAATGGGGTGGAGACTTGGAAATCCCCGTGAGTCAAACCGCTATCCACGCCC
ATTGATGTACTGCCAAAACCGCATCACCATGGTAATAGCGATGACTAATACGTAGATGTACT
GCCAAGTAGGAAAGTCCCATAAGGTCATGTACTGGGCATAATGCCAGGCGGGCCATTTACCG
TCATTGACGTCAATAGGGGGCGTACTTGGCATATGATACACTTGATGTACTGCCAAGTGGGC
AGTTTACCGTAAATACTCCACCCATTGACGTCAATGGAAAGTCCCTATTGGCGTTACTATGG
GAACATACGTCATTATTGACGTCAATGGGCGGGGGTCGTTGGGCGGTCAGCCAGGCGGGCCA
TTTACCGTAAGTTATGTAACGCGGAACTCCATATATGGGCTATGAACTAATGACCCCGTAAT
TGATTACTATTAATAACTAGTCAATAATCAATGTCAACGCGTATATCTGGCCCGTACATCGC
GAAT

DonorD2.4 (pMDS-GOI-TETon- tTS):

CGATACTACGATACTAGTGCCCTTTCGTCTTCAAGAATTCCTCGAGTTTACTCCCTATCAGT
GATAGAGAACGTATGAAGAGTTTACTCCCTATCAGTGATAGAGAACGTATGCAGACTTTACT
CCCTATCAGTGATAGAGAACGTATAAGGAGTTTACTCCCTATCAGTGATAGAGAACGTATGA
CCAGTTTACTCCCTATCAGTGATAGAGAACGTATCTACAGTTTACTCCCTATCAGTGATAGA
GAACGTATATCCAGTTTACTCCCTATCAGTGATAGAGAACGTATAAGCTTTAGGCGTGTACG
GTGGGCGCCTATAAAAGCAGAGCTCGTTTAGTGAACCGTCAGATCGCCTGGAGCAATTCCAC
AACACTTTTGTCTTATACCAACTTTCCGTACCACTTCCTACCCTCGTAAACCCGGGATCTCG
AGCCATGGTGCTAGCAGCTGATGCATAGCATGCGGTACCTAATTCACTCCTCAGGTGCAGGC
TGCCTATCAGAAGGTGGTGGCTGGTGTGGCTAATGCCCTGGCTCACAAATACCACTGAGATC
TTTTTCCCTCTGCCAAAAATTATGGGGACATCATGAAGCCCCTTGAGCATCTGACTTCTGGC
TAATAAAGGAAATTTATTTTCATTGCAATAGTGTGTTGGAATTTTTTGTGTCTCTCACTCGG
AAGGACATATGGGAGGGCAAATCATTTAAAACATCAGAATGAGTATTTGGTTTAGAGTTTGG
CAACATATGCCATATGCTGGCTGCCATGAACAAAGGTGGCTATAAAGAGGTCATCAGTATAT
GAAACAGCCCCCTGCTGTCCATTCCTTATTCCATAGAAAAGCCTTGACTTGAGGTTAGATTT
TTTTTATATTTTGTTTTGTGTTATTTTTTTCTTTAACATCCCTAAAATTTTCCTTACATGTT
TTACTAGCCAGATTTTTCCTCCTCTCCTGACTACTCCCAGTCATAGCTGTCCCTCTTCTCTT
ATGAAGATCCCTCGACGTTTAAACCCATGTGCCTGGCAGATAACTTCGTATAATGTATGCTA
TACGAAGTTATGGTACGTACTAAGCTCTCATGTTTCACGTACTAAGCTCTCATGTTTAACGT
ACTAAGCTCTCATGTTTAACGAACTAAACCCTCATGGCTAACGTACTAAGCTCTCATGGCTA
ACGTACTAAGCTCTCATGTTTCACGTACTAAGCTCTCATGTTTGAACAATAAAATTAATATA

AATCAGCAACTTAAATAGCCTCTAAGGTTTTAAGTTTTATAAGAAAAAAAAGAATATATAAG
GCTTTTAAAGCTTTTAAGGTTTAACGGTTGTGGACAACAAGCCAGGGATGTAACGCACTGAG
AAGCCCTTAGAGCCTCTCAAAGCAATTTTGAGTGACACAGGAACACTTAACGGCTGACATAA
TTCAGCTTCACGCTGCCGCAAGCACTCAGGGCGCAAGGGCTGCTAAAGGAAGCGGAACACGT
AGAAAGCCAGTCCGCAGAAACGGTGCTGACCCCGGATGAATGTCAGCTGGGAGGCAGAATAA
ATGATCATATCGTCAATTATTACCTCCACGGGGAGAGCCTGAGCAAACTGGCCTCAGGCATT
TGAGAAGCACACGGTCACACTGCTTCCGGTAGTCAATAAACCGGTAAGTAGCGTATGCGCTC
ACGCAACTGGTCCAGAACCTTGACCGAACGCAGCGGTGGTAACGGCGCAGTGGCGGTTTTCA
TGGCTTGTTATGACTGTTTTTTTGGGGTACAGTCTATGCCTCGGGCATCCAAGCAGCAAGCG
CGTTACGCCGTGGGTCGATGTTTGATGTTATGGAGCAGCAACGATGTTACGCAGCAGGGCAG
TCGCCCTAAAACAAAGTTAAACATCATGAGGGAAGCGGTGATCGCCGAAGTATCGACTCAAC
TATCAGAGGTAGTTGGCGTCATCGAGCGCCATCTCGAACCGACGTTGCTGGCCGTACATTTG
TACGGCTCCGCAGTGGATGGCGGCCTGAAGCCACACAGTGATATTGATTTGCTGGTTACGGT
GACCGTAAGGCTTGATGAAACAACGCGGCGAGCTTTGATCAACGACCTTTTGGAAACTTCGG
CTTCCCCTGGAGAGAGCGAGATTCTCCGCGCTGTAGAAGTCACCATTGTTGTGCACGACGAC
ATCATTCCGTGGCGTTATCCAGCTAAGCGCGAACTGCAATTTGGAGAATGGCAGCGCAATGA
CATTCTTGCAGGTATCTTCGAGCCAGCCACGATCGACATTGATCTGGCTATCTTGCTGACAA
AAGCAAGAGAACATAGCGTTGCCTTGGTAGGTCCAGCGGCGGAGGAACTCTTTGATCCGGTT
CCTGAACAGGATCTATTTGAGGCGCTAAATGAAACCTTAACGCTATGGAACTCGCCGCCCGA
CTGGGCTGGCGATGAGCGAAATGTAGTGCTTACGTTGTCCCGCATTTGGTACAGCGCAGTAA
CCGGCAAAATCGCGCCGAAGGATGTCGCTGCCGACTGGGCAATGGAGCGCCTGCCGGCCCAG
TATCAGCCCGTCATACTTGAAGCTAGACAGGCTTATCTTGGACAAGAAGAAGATCGCTTGGC
CTCGCGCGCAGATCAGTTGGAAGAATTTGTCCACTACGTGAAAGGCGAGATCACCAAGGTAG
TCGGCAAATAATGTCTAACAATTCGTTCAAGCCGACGGATCTATGTCGGGTGCGGAGAAAGA
GGTAATGAAATGGCACCTAGGGGTTATGATAGTTATTGCTCAGCGGTGGCAGCAGCCAACTC
AGCTTCCTTTCGGGCCAAGCTTGGTCGAGCTGGATACTTCCCGTCCGCCAGGGGGACATGCC
GGCGATGCTGAAGGTCGCGCGCATTCCCGATGAAGAGGCCGGTTACCGCCTGTTGACCTGGT
GGGACGGGCAGGGCGCCGCCCGAGTCTTCGCCTCGGCGGCGGGCGCTCTGCTCATGGAGCGC
GCGTCCGGGGCCGGGGACCTTGCACAGATAGCGTGGTCCGGCCAGGACGACGAGGCTTGCAG
GATCATAATCAGCCATACCACATTTGTAGAGGTTTTACTTGCTTTAAAAAACCTCCCACACC
TCCCCCTGAACCTGAAACATAAAATGAATGCAATTGTTGTTGTTAACTTGTTTATTGCAGCT
TATAATGGTTACAAATAAAGCAATAGCATCACAAATTTCACAAATAAAGCATTTTTTTCACT
GCATTCTAGTTGTGGTTTGTCCAAACTCATCAATGTATCTTATCATGTCTGGATCCTTACTT
AGTTAATCGATTTACCAGGGATCCTCTCCTTGCTGCAACAGGGAGATCACCTTTGGTTTGGT
AAACAGGAATCCTGCCATGGAGGCCAGGTTGCTGTAATTCTCCAGCATCACCTCACGGTACA
GGCTTCTCTGAGACAGATCCAGCTTCTTCCACTCGTCCCGAGTAAAGAGCACAGCCACATCT
TCAAATGTCACTGACACTGCTAGCTTTCTCTTCTTTTTTGGTTTATTACCATCCTCAATGGG
TGTATGCTTTTTTTTCATCTAACTGTGCAGAAAATCCAAATATTAATGATTTCAGCCCGAAAT
GGAAGGCCATTTCAGCAGAGGTTCTCGCCTGAATATTAAATGCCTCTTGTAATAATGGTGGT
GCAGCGTCTATCACATGATTATTTTCTATCTGGTTTGTTGCTTGCTCCTCTAATACTGCACC
CAACGTAAAATGGCTGATAGATTGCAGAATGAAAAGAGCCTCCTCGACCGAAAACCCTGCAT
CGCATAGACAGCGTAGTTGCGCCTCTGCTTGTTCAAACTGGGGGGGCGTAGGAGAGGTCCCT
ATATGCAATCGGGCTCCATCACGATGGACCAGTAATGCTTTACGGAAACTCAGAGCATTTTC
CTGGAGAAACTGCTGCCAACTCTCAGTCGGTAACGGTGCTGAACGGGTGTGATGCTTCGCCA

GTATTGCCTCTGAAAGCATGTTCATAAGAGTCTGCTTGTTGCGCACGTGCCAATACAATGTA
GGCTGCTCTACACCTAGCTTCTGGGCGAGTTTACGGGTTGTTAAACCTTCGATTCCGACCTC
ATTAAGCAGCTCTAATGCGCTGTTAATCACTTTACTTTTATCTAATCTAGACATGGTTGTGG
CCATATTATCATCGTGTTTTTCAAAGGAAAACCACGTCCCCGTGGTTCGGGGGGCCTAGACG
TTTTTTTAACCTCGACTAAACACATGTAAAGCATGTGTACCGAGGCCCCAGATCAGATCCCA
TACAATGGGGTACCTTCTGGGCATCCTTCAGCCCCTTGTTGAATACGCTTGAGGAGAGCCAT
TTGACTCTTTCCACAACTATCCAACTCACAACGTGGCACTGGGGTTGTGCCGCCTTTGCAGG
TGTATCTTATACACGTGGCTTTTGGCCGCAGAGGCACCTGTCGCCAGGTGGGGGGTTCCGCT
GCCTGCAAAGGGTCGCTACAGACGTTGTTTGTCTTCAAGAAGCTTCCAGAGGAACTGCTTCC
TTCACGACATTCAACAGACCTTGCATTCCTTTGGCGAGAGGGGAAAGACCCCTAGGAATGCT
CGTCAAGAAGACAGGGCCAGGTTTCCGGGCCCTCACATTGCCAAAAGACGGCAATATGGTGG
AAAATAACATATAGACAAACGCACACCGGCCTTATTCCAAGCGGCTTCGGCCAGTAACGTTA
GGGGGGGGGGAGGGAGAGGGGCGGATCCCGGGCCCGCGGTACCGTCGACTGCAGATTACCCG
GGGAGCATGTCAAGGTCAAAATCGTCAAGAGCGTCAGCAGGCAGCATATCAAGGTCAAAGTC
GTCAAGGGCATCGGCTGGGAGCATGTCTAAGTCAAAATCGTCAAGGGCGTCGGTCGGCCCGC
CGCTTTCGCACTTTAGCTGTTTCTCCAGGCCACATATGATTAGTTCCAGGCCGAAAAGGAAG
GCAGGTTCGGCTCCCTGCCGGTCGAACAGCTCAATTGCTTGTTTCAGAAGTGGGGGCATAGA
ATCGGTGGTAGGTGTCTCTCTTTCCTCTTTTGCTACTTGATGCTCCTGTTCCTCCAATACGC
AGCCCAGTGTAAAGTGGCCCACGGCGGACAGAGCGTACAGTGCGTTCTCCAGGGAGAAGCCT
TGCTGACACAGGAACGCGAGCTGATTTTCCAGGGTTTCGTACTGTTTCTCTGTTGGGCGGGT
GCCGAGATGCACTTTAGCCCCGTCGCGATGTGAGAGGAGAGCACAGCGGTATGACTTGGCGT
TGTTCCGCAGAAAGTCTTGCCATGACTCGCCTTCCAGGGGGCAGGAGTGGGTATGATGCCTG
TCCAGCATCTCGATTGGCAGGGCATCGAGCAGGGCCCGCTTGTTCTTCACGTGCCAGTACAG
GGTAGGCTGCTCAACTCCCAGCTTTTGAGCGAGTTTCCTTGTCGTCAGGCCTTCGATACCGA
CTCCATTGAGTAATTCCAGAGCAGAGTTTATGACTTTGCTCTTGTCCAGTCTAGACATGGTG
AATTCCGCGCGCTTCGGACCGGGATCCACTAGCCAGCTTGGGTCTCCCTATAGTGAGTCGTA
TTAATTTCGATAAGCCAGTAAGCAGTGGGTTCTCTAGTTAGCCAGAGAGCTCTGCTTATATA
GACCTCCCACCGTACACGCCTACCGCCCATTTGCGTCAATGGGGCGGAGTTGTTACGACATT
TTGGAAAGTCCCGTTGATTTTGGTGCCAAAACAAACTCCCATTGACGTCAATGGGGTGGAGA
CTTGGAAATCCCCGTGAGTCAAACCGCTATCCACGCCCATTGATGTACTGCCAAAACCGCAT
CACCATGGTAATAGCGATGACTAATACGTAGATGTACTGCCAAGTAGGAAAGTCCCATAAGG
TCATGTACTGGGCATAATGCCAGGCGGGCCATTTACCGTCATTGACGTCAATAGGGGGCGTA
CTTGGCATATGATACACTTGATGTACTGCCAAGTGGGCAGTTTACCGTAAATACTCCACCCA
TTGACGTCAATGGAAAGTCCCTATTGGCGTTACTATGGGAACATACGTCATTATTGACGTCA
ATGGGCGGGGGTCGTTGGGCGGTCAGCCAGGCGGGCCATTTACCGTAAGTTATGTAACGCGG
AACTCCATATATGGGCTATGAACTAATGACCCCGTAATTGATTACTATTAATAACTAGTCAA
TAATCAATGTCAACGCGTATATCTGGCCCGTACATCGCGAAT

Acceptor A (pACEMam3):

ACCGGTTGACTTGGGTCAACTGTCAGACCAAGTTTACTCATATATACTTTAGATTGATTTAA
AACTTCATTTTTAATTTAAAAGGATCTAGGTGAAGATCCTTTTTGATAATCTCATGACCAAA
ATCCCTTAACGTGAGTTTTCGTTCCACTGAGCGTCAGACCCCGTAGAAAAGATCAAAGGATC

TTCTTGAGATCCTTTTTTTCTGCGCGTAATCTGCTGCTTGCAAACAAAAAAACCACCGCTAC
CAGCGGTGGTTTGTTTGCCGGATCAAGAGCTACCAACTCTTTTTCCGAAGGTAACTGGCTTC
AGCAGAGCGCAGATACCAAATACTGTTCTTCTAGTGTAGCCGTAGTTAGGCCACCACTTCAA
GAACTCTGTAGCACCGCCTACATACCTCGCTCTGCTAATCCTGTTACCAGTGGCTGCTGCCA
GTGGCGATAAGTCGTGTCTTACCGGGTTGGACTCAAGACGATAGTTACCGGATAAGGCGCAG
CGGTCGGGCTGAACGGGGGGTTCGTGCACACAGCCCAGCTTGGAGCGAACGACCTACACCGA
ACTGAGATACCTACAGCGTGAGCTATGAGAAAGCGCCACGCTTCCCGAAGGGAGAAAGGCGG
ACAGGTATCCGGTAAGCGGCAGGGTCGGAACAGGAGAGCGCACGAGGGAGCTTCCAGGGGGA
AACGCCTGGTATCTTTATAGTCCTGTCGGGTTTCGCCACCTCTGACTTGAGCGTCGATTTTT
GTGATGCTCGTCAGGGGGGCGGAGCCTATGGAAAAACGCCAGCAACGCGGCCTTTTTACGGT
TCCTGGCCTTTTGCTGGCCTTTTGCTCACATGTTCTTTCCTGCGTTATCCCCTGATTGACTT
GGGTCGCTCTTCCTGTGGATGCGCACAGACATGATAAGATACATTGATGAGTTTGGACAAAC
CACAACTAGAATGCAGTGAAAAAAATGCTTTATTTGTGAAATTTGTGATGCTATTGCTTTAT
TTGTAACCATTATAAGCTGCAATAAACAAGTTGGGGTGGGCGAAGAACTCCAGCATGAGATC
CCCGCGCTGGAGGATCATCCAGCCGGCGTCCCGGAAAACGATTCCGAAGCCCAACCTTTCAT
AGAAGGCGGCGGTGGAATCGAAATCTCGTAGCACGTGTCAGTCCTGCTCCTCGGCCACGAAG
TGCACGCAGTTGCCGGCCGGGTCGCGCAGGGCGAACTCCCGCCCCCACGGCTGCTCGCCGAT
CTCGGTCATGGCCGGCCCGGAGGCGTCCCGGAAGTTCGTGGACACGACCTCCGACCACTCGG
CGTACAGCTCGTCCAGGCCGCGCACCCACACCCAGGCCAGGGTGTTGTCCGGCACCACCTGG
TCCTGGACCGCGCTGATGAACAGGGTCACGTCGTCCCGGACCACACCGGCGAAGTCGTCCTC
CACGAAGTCCCGGGAGAACCCGAGCCGGTCGGTCCAGAACTCGACCGCTCCGGCGACGTCGC
GCGCGGTGAGCACCGGAACGGCACTGGTCAACTTGGCCATGGTTTAGTTCCTCACCTTGTCG
TATTATACTATGCCGATATACTATGCCGATGATTAATTGTCAACACGTGCTGATCAGATCCG
AAAATGGATATACAAGCTCCCGGGAGCTTTTTGCAAAAGCCTAGGCCTCCAAAAAAGCCTCC
TCACTACTTCTGGAATAGCTCAGAGGCAGAGGCGGCCTCGGCCTCTGCATAAATAAAAAAAA
TTAGTCAGCCATGGGGCGGAGAATGGGCGGAACTGGGCGGAGTTAGGGGCGGGATGGGCGGA
GTTAGGGGCGGGACTATGGTTGCTGACTAATTGAGATGCATGCTTTGCATACTTCTGCCTGC
TGGGGAGCCTGGGGACTTTCCACACCTGGTTGCTGACTAATTGAGATGCATGCTTTGCATAC
TTCTGCCTGCCTGGAAGTTCCTATACTTTCTAGAGAATAGGAACTTCGGAATAGGAACTTCA
TTACCCTGTTATCCCTACCCATAATACCCATAATAGCTGTTTGCCAGCAGTGAAAAAAATGC
TTTATTTGTGAAATTTGTGATGCTATTGCTTTATTTGTAACCATTATAAGCTGCAATAAACA
AGTTAACAACAACAATTGCATTCATTTTATGTTTCAGGTTCAGGGGGAGGTGTGGGAGGTTT
TTTAAAGCAAGTAAAACCTCTACAAATGTGGTATGGCTGATTATGATCCTCTAGAACTCTAT
TCCTTTGCCCTCGGACGAGTGCTGGGGCGTCGGTTTCCACTATCGGCGAGTACTTCTACACA
GCCATCGGTCCAGACGGCCGCGCTTCTGCGGGCGATTTGTGTACGCCCGACAGTCCCGGCTC
CGGATCGGACGATTGCGTCGCATCGACCCTGCGCCCAAGCTGCATCATCGAAATTGCCGTCA
ACCAAGCTCTGATAGAGTTGGTCAAGACCAATGCGGAGCATATACGCCCGGAGCCGCGGCGA
TCCTGCAAGCTCCGGATGCCTCCGCTCGAAGTAGCGCGTCTGCTGCTCCATACAAGCCAACC
ACGGCCTCCAGAAGAAGATGTTGGCGACCTCGTATTGGGAATCCCCGAACATCGCCTCGCTC
CAGTCAATGACCGCTGTTATGCGGCCATTGTCCGTCAGGACATTGTTGGAGCCGAAATCCGC
GTGCACGAGGTGCCGGACTTCGGGGCAGTCCTCGGCCCAAAGCATCAGCTCATCGAGAGCCT
GCGCGACGGACGCACTGACGGTGTCGTCCATCACAGTTTGCCAGTGATACACATGGGGATCA
GCAATCGCGCATATGAAATCACGCCATGTAGTGTATTGACCGATTCCTTGCGGTCCGAATGG
GCCGAACCCGCTCGTCTGGCTAAGATCGGCCGCAGCGATCGCATCCATGGCCTCCGCGACCG

GCTGCAGAACAGCGGGCAGTTCGGTTTCAGGCAGGTCTTGCAACGTGACACCCTGTGCACGG
CGGGAGATGCAATAGGTCAGGCTCTCGCTGAATTCCCCAATGTCAAGCACTTCCGGAATCGG
GAGCGCGGCCGATGCAAAGTGCCGATAAACATAACGATCTTTGTAGAAACCATCGGCGCAGC
TATTTACCCGCAGGACATATCCACGCCCTCCTACATCGAAGCTGAAAGCACGAGATTCTTCG
CCCTCCGAGAGCTGCATCAGGTCGGAGACGCTGTCGAACTTTTCGATCAGAAACTTCTCGAC
AGACGTCGCGGTGAGTTCAGGCTTTTTCATGGAAGCTTTTTGCAAAAGCCTAGGCCTCCAAA
AAAGCCTCCTCACTACTTCTGGAATAGCTCAGAGGCCGAGGCGGCCTCGGCCTCTGCATAAA
TAAAAAAAATTAGTCAGCCATGGGGCGGAGAATGGGCGGAACTGGGCGGAGTTAGGGGCGGG
ATGGGCGGAGTTAGGGGCGGGACTATGGTTGCTGACTAATTGAGATGCATGCTTTGCATACT
TCTGCCTGCTGGGGAGCCTGGGGACTTTCCACACCTGGTTGCTGACTAATTGAGATGCATGC
TTTGCATACTTCTGCCTGCTGGGGAGCCTGGGGACTTTCCACACGGTGTGCGTCACCCGGCA
ACCTTGGGCAGCAGCGAAGTCGCCATAACTTCGTATAGCATACATTATACGAAGTTATCTGT
AACTATAACGGTCCTAAGGTAGCGAGTTTAAACGTCGAGGGATCTTCATAAGAGAAGAGGGA
CAGCTATGACTGGGAGTAGTCAGGAGAGGAGGAAAAATCTGGCTAGTAAAACATGTAAGGAA
AATTTTAGGGATGTTAAAGAAAAAAATAACACAAAACAAAATATAAAAAAAATCTAACCTCA
AGTCAAGGCTTTTCTATGGAATAAGGAATGGACAGCAGGGGGCTGTTTCATATACTGATGAC
CTCTTTATAGCCACCTTTGTTCATGGCAGCCAGCATATGGCATATGTTGCCAAACTCTAAAC
CAAATACTCATTCTGATGTTTTAAATGATTTGCCCTCCCATATGTCCTTCCGAGTGAGAGAC
ACAAAAAATTCCAACACACTATTGCAATGAAAATAAATTTCCTTTATTAGCCAGAAGTCAGA
TGCTCAAGGGGCTTCATGATGTCCCCATAATTTTTGGCAGAGGGAAAAAGATCTCAGTGGTA
TTTGTGAGCCAGGGCATTAGCCACACCAGCCACCACCTTCTGATAGGCAGCCTGCACCTGAG
GAGTGAATTAGGTACCTCATTAGTCGACTGCAGAATTCGAAGCTTGAGCTCGAGATCTGAGT
CCGGACTTGTACAGCTCGTCCATGCCGAGAGTGATCCCGGCGGCGGTCACGAACTCCAGCAG
GACCATGTGATCGCGCTTCTCGTTGGGGTCTTTGCTCAGGGCGGACTGGTAGCTCAGGTAGT
GGTTGTCGGGCAGCAGCACGGGGCCGTCGCCGATGGGGGTGTTCTGCTGGTAGTGGTCGGCG
AGCTGCACGCTGCCGTCCTCGATGTTGTGGCGGATCTTGAAGTTCACCTTGATGCCGTTCTT
CTGCTTGTCGGCCATGATATAGACGTTGTGGCTGTTGTAGTTGTACTCCAGCTTGTGCCCCA
GGATGTTGCCGTCCTCCTTGAAGTCGATGCCCTTCAGCTCGATGCGGTTCACCAGGGTGTCG
CCCTCGAACTTCACCTCGGCGCGGGTCTTGTAGTTGCCGTCGTCCTTGAAGAAGATGGTGCG
CTCCTGGACGTAGCCTTCGGGCATGGCGGACTTGAAGAAGTCGTGCTGCTTCATGTGGTCGG
GGTAGCGGGCGAAGCACTGCAGGCCGTAGCCGAAGGTGGTCACGAGGGTGGGCCAGGGCACG
GGCAGCTTGCCGGTGGTGCAGATGAACTTCAGGGTCAGCTTGCCGTAGGTGGCATCGCCCTC
GCCCTCGCCGGACACGCTGAACTTGTGGCCGTTTACGTCGCCGTCCAGCTCGACCAGGATGG
GCACCACCCCGGTGAACAGCTCCTCGCCCTTGCTCACCATGGCTCGAGATCCCGGGTGATCA
AGTCTTCGGTGGCCTGAGACGGCCGCAATTCTTTGCCAAAATGATGAGACAGCACAACAACC
AGCACGTTGCCCAGGAGCTGTAGGAAAAAGAAGAAGGCATGAACATGGTTAGCAGAGGCTCT
AGCAGCCGCCGGTCACACGCCAGAAGCCGAACCCCGCCCTGCCCCGTCCCCCCCGAAGGCAG
CCGTCCCCCTGCGGCAGCCCCGAGGCTGGAGATGGAGAAGGGGACGGCGGCGCGGCGACGCA
CGAAGGCCCTCCCCGCCCATTTCCTTCCTGCCGGCGCCGCACCGCTTCGCCCGCGCCCGCTA
GAGGGGGTGCGGCGGCGCCTCCCAGATTTCGGCTCCGCCAGATTTGGGACAAAGGAAGTCCC
TGCGCCCTCTCGCACGATTACCATAAAAGGCAATGGCTGCGGCTCGCCGCGCCTCGACAGCC
GCCGGCGCTCCGGGGCCGCCGCGCCCCTCCCCCGAGCCCTCCCCGGCCCGAGGCGGCCCCGC
CCCGCCCGGCACCCCCACCTGCCGCCACCCCCCGCCCGGCACGGCGAGCCCCGCGCCACGCC
CCGCACGGAGCCCCGCACCCGAAGCCGGGCCGTGCTCAGCAACTCGGGGAGGGGGTGCAGG

GGGGGGTTACAGCCCGACCGCCGCGCCCACACCCCCTGCTCACCCCCCCACGCACACACCCC
GCACGCAGCCTTTGTTCCCCTCGCAGCCCCCCCGCACCGCGGGGCACCGCCCCCGGCCGCGC
TCCCCTCGCGCACACGCGGAGCGCACAAAGCCCCGCGCCGCGCCCGCAGCGCTCACAGCCGC
CGGGCAGCGCGGGCCGCACGCGGCGCTCCCCACGCACACACACACGCACGCACCCCCCGAGC
CGCTCCCCCCCGCACAAAGGGCCCTCCCGGAGCCCTTTAAGGCTTTCACGCAGCCACAGAAA
AGAAACGAGCCGTCATTAAACCAAGCGCTAATTACAGCCCGGAGGAGAAGGGCCGTCCCGCC
CGCTCACCTGTGGGAGTAACGCGGTCAGTCAGAGCCGGGGCGGGCGGCGCGAGGCGGCGCGG
AGCGGGGCACGGGGCGAAGGCAACGCAGCGACTCCCGCCCGCCGCGCGCTTCGCTTTTTATA
GGGCCGCCGCCGCCGCCGCCTCGCCATAAAAGGAAACTTTCGGAGCGCGCCGCTCTGATTGG
CTGCCGCCGCACCTCTCCGCCTCGCCCCGCCCCGCCCCTCGCCCCGCCCCGCCCCGCCTGGC
GCGCGCCCCCCCCCCCCCCGCCCCCATCGCTGCACAAAATAATTAAAAAATAAATAAATACA
AAATTGGGGGTGGGGAGGGGGGGGAGATGGGGAGAGTGAAGCAGAACGTGGGGCTCACCTCG
ACCCATGAGTAATAGCGATGACTAATACGTAGATGTACTGCCAAGTAGGAAAGTCCCATAAG
GTCATGTACTGGGCATAATGCCAGGCGGGCCATTTACCGTCATTGACGTCAATAGGGGGCGT
ACTTGGCATATGATACACTTGATGTACTGCCAAGTGGGCAGTTTACCGTAAATAGTCCACCC
ATTGACGTCAATGGAAAGTCCCTATTGGCGTTACTATGGGAACATACGTCATTATTGACGTC
AATGGGCGGGGGTCGTTGGGCGGTCAGCCAGGCGGGCCATTTACCGTAAGTTATGTAACGCG
GAACTCCATATATGGGCTATGAACTAATGACCCCGTAATTGATTACTATTAATAACGTATAC
TAGTATCGTAGTATCGATCCATCTAATTGGAACCAGATAAGTGAAATCTAGTTCCAAACTAT
TTTGTCATTTTTAATTTTCGTATTAGCTTACGACGCTACACCCAGTTCCCATCTATTTTGTC
ACTCTTCCCTAAATAATCCTTAAAAACTCCATTTCCACCCCTCCCAGTTCCCAACTATTTTG
TCCGCCCACA

Sequences for pACEMAM4 (PiggyBac compatible):

Backbone pACEMAM3:

CAGGCAGGCAGAAGTATGCAAAGCATGCATCTCAATTAGTCAGCAACCAGGTGTGGAAAGTC
CCCAGGCTCCCCAGCAGGCAGAAGTATGCAAAGCATGCATCTCAATTAGTCAGCAACCATAG
TCCCGCCCCTAACTCCGCCCATCCCGCCCCTAACTCCGCCCAGTTCCGCCCATTCTCCGCCC
CATGGCTGACTAATTTTTTTTATTTATGCAGAGGCCGAGGCCGCCTCTGCCTCTGAGCTATT
CCAGAAGTAGTGAGGAGGCTTTTTTGGAGGCCTAGGCTTTTGCAAAAAGCTCCCGGGAGCTT
GTATATCCATTTTCGGATCTGATCAGCACGTGTTGACAATTAATCATCGGCATAGTATATCG
GCATAGTATAATACGACAAGGTGAGGAACTAAACCATGGCCAAGTTGACCAGTGCCGTTCCG
GTGCTCACCGCGCGCGACGTCGCCGGAGCGGTCGAGTTCTGGACCGACCGGCTCGGGTTCTC
CCGGGACTTCGTGGAGGACGACTTCGCCGGTGTGGTCCGGGACGACGTGACCCTGTTCATCA
GCGCGGTCCAGGACCAGGTGGTGCCGGACAACACCCTGGCCTGGGTGTGGGTGCGCGGCCTG
GACGAGCTGTACGCCGAGTGGTCGGAGGTCGTGTCCACGAACTTCCGGGACGCCTCCGGGCC
GGCCATGACCGAGATCGGCGAGCAGCCGTGGGGGCGGGAGTTCGCCCTGCGCGACCCGGCCG
GCAACTGCGTGCACTTCGTGGCCGAGGAGCAGGACTGACACGTGCTACGAGATTTCGATTCC
ACCGCCGCCTTCTATGAAAGGTTGGGCTTCGGAATCGTTTTCCGGGACGCCGGCTGGATGAT
CCTCCAGCGCGGGGATCTCATGCTGGAGTTCTTCGCCCACCCCAACTTGTTTATTGCAGCTT
ATAATGGTTACAAATAAAGCAATAGCATCACAAATTTCACAAATAAAGCATTTTTTTCACTG
CATTCTAGTTGTGGTTTGTCCAAACTCATCAATGTATCTTATCATGTCTGTGCGCATCCACA
GGAAGAGCGACCCAAGTCAATCAGGGGATAACGCAGGAAAGAACATGTGAGCAAAAGGCCAG
CAAAAGGCCAGGAACCGTAAAAAGGCCGCGTTGCTGGCGTTTTTCCATAGGCTCCGCCCCCC
TGACGAGCATCACAAAAATCGACGCTCAAGTCAGAGGTGGCGAAACCCGACAGGACTATAAA
GATACCAGGCGTTTCCCCCTGGAAGCTCCCTCGTGCGCTCTCCTGTTCCGACCCTGCCGCTT
ACCGGATACCTGTCCGCCTTTCTCCCTTCGGGAAGCGTGGCGCTTTCTCATAGCTCACGCTG
TAGGTATCTCAGTTCGGTGTAGGTCGTTCGCTCCAAGCTGGGCTGTGTGCACGAACCCCCCG
TTCAGCCCGACCGCTGCGCCTTATCCGGTAACTATCGTCTTGAGTCCAACCCGGTAAGACAC
GACTTATCGCCACTGGCAGCAGCCACTGGTAACAGGATTAGCAGAGCGAGGTATGTAGGCGG
TGCTACAGAGTTCTTGAAGTGGTGGCCTAACTACGGCTACACTAGAAGAACAGTATTTGGTA
TCTGCGCTCTGCTGAAGCCAGTTACCTTCGGAAAAAGAGTTGGTAGCTCTTGATCCGGCAAA
CAAACCACCGCTGGTAGCGGTGGTTTTTTTGTTTGCAAGCAGCAGATTACGCGCAGAAAAAA
AGGATCTCAAGAAGATCCTTTGATCTTTTCTACGGGGTCTGACGCTCAGTGGAACGAAAACT
CACGTTAAGGGATTTTGGTCATGAGATTATCAAAAAGGATCTTCACCTAGATCCTTTTAAAT
TAAAAATGAAGTTTTAAATCAATCTAAAGTATATATGAGTAAACTTGGTCTGACAGTTGACC
CAAGTCAACCGGTTGTGGGCGGACAAAATAGTTGGGAACTGGGAGGGGTGGAAATGGAGTTT
TTAAGGATTATTTAGGGAAGAGTGACAAAATAGATGGGAACTGGGTGTAGCGTCGTAAGCTA
ATACGAAAATTAAAAATGACAAAATAGTTTGGAACTAGATTTCACTTATCTGGTTCCAATTA
GATGGATCGATACTACGATACTAGTATACGTTATTAATAGTAATCAATTACGGGGTCATTAG
TTCATAGCCCATATATGGAGTTCCGCGTTACATAACTTACGGTAAATGGCCCGCCTGGCTGA
CCGCCCAACGACCCCCGCCCATTGACGTCAATAATGACGTATGTTCCCATAGTAACGCCAAT
AGGGACTTTCCATTGACGTCAATGGGTGGACTATTTACGGTAAACTGCCCACTTGGCAGTAC
ATCAAGTGTATCATATGCCAAGTACGCCCCCTATTGACGTCAATGACGGTAAATGGCCCGCC
TGGCATTATGCCCAGTACATGACCTTATGGGACTTTCCTACTTGGCAGTACATCTACGTATT
AGTCATCGCTATTACTCATGGGTCGAGGTGAGCCCCACGTTCTGCTTCACTCTCCCCATCTC

CCCCCCCTCCCCACCCCCAATTTTGTATTTATTTATTTTTTAATTATTTTGTGCAGCGATGG
GGGCGGGGGGGGGGGGGGCGCGCGCCAGGCGGGGCGGGGCGGGGCGAGGGGCGGGGCGGGGC
GAGGCGGAGAGGTGCGGCGGCAGCCAATCAGAGCGGCGCGCTCCGAAAGTTTCCTTTTATGG
CGAGGCGGCGGCGGCGGCGGCCCTATAAAAAGCGAAGCGCGCGGCGGGCGGGAGTCGCTGCG
TTGCCTTCGCCCCGTGCCCCGCTCCGCGCCGCCTCGCGCCGCCCGCCCCGGCTCTGACTGAC
CGCGTTACTCCCACAGGTGAGCGGGCGGGACGGCCCTTCTCCTCCGGGCTGTAATTAGCGCT
TGGTTTAATGACGGCTCGTTTCTTTTCTGTGGCTGCGTGAAAGCCTTAAAGGGCTCCGGGAG
GGCCCTTTGTGCGGGGGGGAGCGGCTCGGGGGGTGCGTGCGTGTGTGTGTGCGTGGGGAGCG
CCGCGTGCGGCCCGCGCTGCCCGGCGGCTGTGAGCGCTGCGGGCGCGGCGCGGGGCTTTGTG
CGCTCCGCGTGTGCGCGAGGGGAGCGCGGCCGGGGGCGGTGCCCCGCGGTGCGGGGGGGCTG
CGAGGGGAACAAAGGCTGCGTGCGGGGTGTGTGCGTGGGGGGGTGAGCAGGGGGTGTGGGCG
CGGCGGTCGGGCTGTAACCCCCCCCTGCACCCCCCTCCCCGAGTTGCTGAGCACGGCCCGGC
TTCGGGTGCGGGGCTCCGTGCGGGGCGTGGCGCGGGGCTCGCCGTGCCGGGCGGGGGGTGGC
GGCAGGTGGGGGTGCCGGGCGGGGCGGGGCCGCCTCGGGCCGGGGAGGGCTCGGGGGAGGGG
CGCGGCGGCCCCGGAGCGCCGGCGGCTGTCGAGGCGCGGCGAGCCGCAGCCATTGCCTTTTA
TGGTAATCGTGCGAGAGGGCGCAGGGACTTCCTTTGTCCCAAATCTGGCGGAGCCGAAATCT
GGGAGGCGCCGCCGCACCCCCTCTAGCGGGCGCGGGCGAAGCGGTGCGGCGCCGGCAGGAAG
GAAATGGGCGGGGAGGGCCTTCGTGCGTCGCCGCGCCGCCGTCCCCTTCTCCATCTCCAGCC
TCGGGGCTGCCGCAGGGGGACGGCTGCCTTCGGGGGGGACGGGGCAGGGCGGGGTTCGGCTT
CTGGCGTGTGACCGGCGGCTGCTAGAGCCTCTGCTAACCATGTTCATGCCTTCTTCTTTTTC
CTACAGCTCCTGGGCAACGTGCTGGTTGTTGTGCTGTCTCATCATTTTGGCAAAGAATTGCG
GCCGTCTCAGGCCACCGAAGACTTGATCACCCGGGATCTCGAGCCATGGTGAGCAAGGGCGA
GGAGCTGTTCACCGGGGTGGTGCCCATCCTGGTCGAGCTGGACGGCGACGTAAACGGCCACA
AGTTCAGCGTGTCCGGCGAGGGCGAGGGCGATGCCACCTACGGCAAGCTGACCCTGAAGTTC
ATCTGCACCACCGGCAAGCTGCCCGTGCCCTGGCCCACCCTCGTGACCACCTTCGGCTACGG
CCTGCAGTGCTTCGCCCGCTACCCCGACCACATGAAGCAGCACGACTTCTTCAAGTCCGCCA
TGCCCGAAGGCTACGTCCAGGAGCGCACCATCTTCTTCAAGGACGACGGCAACTACAAGACC
CGCGCCGAGGTGAAGTTCGAGGGCGACACCCTGGTGAACCGCATCGAGCTGAAGGGCATCGA
CTTCAAGGAGGACGGCAACATCCTGGGGCACAAGCTGGAGTACAACTACAACAGCCACAACG
TCTATATCATGGCCGACAAGCAGAAGAACGGCATCAAGGTGAACTTCAAGATCCGCCACAAC
ATCGAGGACGGCAGCGTGCAGCTCGCCGACCACTACCAGCAGAACACCCCCATCGGCGACGG
CCCCGTGCTGCTGCCCGACAACCACTACCTGAGCTACCAGTCCGCCCTGAGCAAAGACCCCA
ACGAGAAGCGCGATCACATGGTCCTGCTGGAGTTCGTGACCGCCGCCGGGATCACTCTCGGC
ATGGACGAGCTGTACAAGTCCGGACTCAGATCTCGAGCTCAAGCTTCGAATTCTGCAGTCGA
CTAATGAGGTACCTAATTCACTCCTCAGGTGCAGGCTGCCTATCAGAAGGTGGTGGCTGGTG
TGGCTAATGCCCTGGCTCACAAATACCACTGAGATCTTTTTCCCTCTGCCAAAAATTATGGG
GACATCATGAAGCCCCTTGAGCATCTGACTTCTGGCTAATAAAGGAAATTTATTTTCATTGC
AATAGTGTGTTGGAATTTTTTGTGTCTCTCACTCGGAAGGACATATGGGAGGGCAAATCATT
TAAAACATCAGAATGAGTATTTGGTTTAGAGTTTGGCAACATATGCCATATGCTGGCTGCCA
TGAACAAAGGTGGCTATAAAGAGGTCATCAGTATATGAAACAGCCCCCTGCTGTCCATTCCT
TATTCCATAGAAAAGCCTTGACTTGAGGTTAGATTTTTTTTATATTTTGTTTTGTGTTATTT
TTTTCTTTAACATCCCTAAAATTTTCCTTACATGTTTTACTAGCCAGATTTTTCCTCCTCTC
CTGACTACTCCCAGTCATAGCTGTCCCTCTTCTCTTATGAAGATCCCTCGACGTTTAAACTC
GCTACCTTAGGACCGTTATAGTTACAGATAACTTCGTATAATGTATGCTATACGAAGTTATG

GCGACTTCGCTGCTGCCCAAGGTTGCCGGGTGACGCACACCGTGTGGAAAGTCCCCAGGCTC
CCCAGCAGGCAGAAGTATGCAAAGCATGCATCTCAATTAGTCAGCAACCAGGTGTGGAAAGT
CCCCAGGCTCCCCAGCAGGCAGAAGTATGCAAAGCATGCATCTCAATTAGTCAGCAACCATA
GTCCCGCCCCTAACTCCGCCCATCCCGCCCCTAACTCCGCCCAGTTCCGCCCATTCTCCGCC
CCATGGCTGACTAATTTTTTTTATTTATGCAGAGGCCGAGGCCGCCTCGGCCTCTGAGCTAT
TCCAGAAGTAGTGAGGAGGCTTTTTTGGAGGCCTAGGCTTTTGCAAAAAGCTTCCATGAAAA
AGCCTGAACTCACCGCGACGTCTGTCGAGAAGTTTCTGATCGAAAAGTTCGACAGCGTCTCC
GACCTGATGCAGCTCTCGGAGGGCGAAGAATCTCGTGCTTTCAGCTTCGATGTAGGAGGGCG
TGGATATGTCCTGCGGGTAAATAGCTGCGCCGATGGTTTCTACAAAGATCGTTATGTTTATC
GGCACTTTGCATCGGCCGCGCTCCCGATTCCGGAAGTGCTTGACATTGGGGAATTCAGCGAG
AGCCTGACCTATTGCATCTCCCGCCGTGCACAGGGTGTCACGTTGCAAGACCTGCCTGAAAC
CGAACTGCCCGCTGTTCTGCAGCCGGTCGCGGAGGCCATGGATGCGATCGCTGCGGCCGATC
TTAGCCAGACGAGCGGGTTCGGCCCATTCGGACCGCAAGGAATCGGTCAATACACTACATGG
CGTGATTTCATATGCGCGATTGCTGATCCCCATGTGTATCACTGGCAAACTGTGATGGACGA
CACCGTCAGTGCGTCCGTCGCGCAGGCTCTCGATGAGCTGATGCTTTGGGCCGAGGACTGCC
CCGAAGTCCGGCACCTCGTGCACGCGGATTTCGGCTCCAACAATGTCCTGACGGACAATGGC
CGCATAACAGCGGTCATTGACTGGAGCGAGGCGATGTTCGGGGATTCCCAATACGAGGTCGC
CAACATCTTCTTCTGGAGGCCGTGGTTGGCTTGTATGGAGCAGCAGACGCGCTACTTCGAGC
GGAGGCATCCGGAGCTTGCAGGATCGCCGCGGCTCCGGGCGTATATGCTCCGCATTGGTCTT
GACCAACTCTATCAGAGCTTGGTTGACGGCAATTTCGATGATGCAGCTTGGGCGCAGGGTCG
ATGCGACGCAATCGTCCGATCCGGAGCCGGGACTGTCGGGCGTACACAAATCGCCCGCAGAA
GCGCGGCCGTCTGGACCGATGGCTGTGTAGAAGTACTCGCCGATAGTGGAAACCGACGCCCC
AGCACTCGTCCGAGGGCAAAGGAATAGAGTTCTAGAGGATCATAATCAGCCATACCACATTT
GTAGAGGTTTTACTTGCTTTAAAAAACCTCCCACACCTCCCCCTGAACCTGAAACATAAAAT
GAATGCAATTGTTGTTGTTAACTTGTTTATTGCAGCTTATAATGGTTACAAATAAAGCAATA
GCATCACAAATTTCACAAATAAAGCATTTTTTTCACTG

Primers to amplify:

pACEMAM3_Fw: 5’TGGGCCCATCCAGGCAGGCAGAAGTATG3’

pACEMAM3_Rev:5’GACCATGATTACGCCACAGTGAAAAAAATGCTTTATT3’

PiggyBac 3’Terminal Repeat from pPB_CAG_EBNXN:

TGGCGTAATCATGGTCATAGCTGTTTCCTGTGTGAAATTGTTATCCGCTCACAATTCCACAC
AACATACGAGCCGGAAGCATAAAGTGTAAAGCCTGGGGTGCCTAATGAGTGAGCTAACTCAC
ATTAATTGCGTTGCGCTCACTGCCCGCTTTCCAGTCGGGAAACCTGTCGTGCCAGCGGATCC
ATTCATGAATGAATTCATGTCGACATACTAGTTAAAAGTTTTGTTACTTTATAGAAGAAATT
TTGAGTTTTTGTTTTTTTTTAATAAATAAATAAACATAAATAAATTGTTTGTTGAATTTATT
ATTAGTATGTAAGTGTAAATATAATAAAACTTAATATCTATTCAAATTAATAAATAAACCTC
GATATACAGACCGATAAAACACATGCGTCAATTTTACGCATGATTATCTTTAACGTACGTCA
CAATATGATTATCTTTCTAGGGTTAA

Primers to amplify:

pPB_CAG Fw1: 5'TGGCGTAATCATGGTCATA3'

pPB_CAG Rv1: 5'CAATGTCAACGCTTAACCCTAGAAAGATAATCAT3'

CMV promotor and mTurqoise2 from p30:

GCGTTGACATTGATTATTGACTAGTTATTAATAGTAATCAATTACGGGGTCATTAGTTCATA
GCCCATATATGGAGTTCCGCGTTACATAACTTACGGTAAATGGCCCGCCTGGCTGACCGCCC
AACGACCCCCGCCCATTGACGTCAATAATGACGTATGTTCCCATAGTAACGCCAATAGGGAC
TTTCCATTGACGTCAATGGGTGGAGTATTTACGGTAAACTGCCCACTTGGCAGTACATCAAG
TGTATCATATGCCAAGTACGCCCCCTATTGACGTCAATGACGGTAAATGGCCCGCCTGGCAT
TATGCCCAGTACATGACCTTATGGGACTTTCCTACTTGGCAGTACATCTACGTATTAGTCAT
CGCTATTACCATGGTGATGCGGTTTTGGCAGTACATCAATGGGCGTGGATAGCGGTTTGACT
CACGGGGATTTCCAAGTCTCCACCCCATTGACGTCAATGGGAGTTTGTTTTGGCACCAAAAT
CAACGGGACTTTCCAAAATGTCGTAACAACTCCGCCCCATTGACGCAAATGGGCGGTAGGCG
TGTACGGTGGGAGGTCTATATAAGCAGAGCTCTCTGGCTAACTAGAGAACCCACTGCTTACT
GGCTTATCGAAATTAATACGACTCACTATAGGGAGACCCAAGCTGGCTAGCGCCGCCACCA
TGGTGTCGAAGGGGGAAGAGTTGTTTACGGGAGTAGTGCCGATTCTCGTAGAGCTTGATGGG
GACGTCAATGGTCACAAGTTTTCGGTATCCGGGGAGGGGGAGGGTGACGCAACATACGGAAA
ACTGACGTTGAAGTTTATCTGTACGACGGGAAAACTTCCGGTGCCGTGGCCCACTTTGGTAA
CAACTCTCTCATGGGGCGTGCAGTGCTTCGCACGCTATCCCGATCACATGAAGCAACATGAC
TTTTTCAAGAGCGCGATGCCGGAGGGGTACGTGCAAGAGAGAACCATCTTCTTCAAGGACGA
CGGAAACTACAAAACACGGGCGGAAGTGAAGTTCGAAGGAGACACACTGGTAAACAGGATCG
AACTGAAGGGGATTGACTTTAAGGAAGATGGTAACATCCTTGGACATAAGCTGGAGTATAAC
TACTTTTCCGATAATGTCTATATCACGGCGGATAAGCAGAAAAACGGAATCAAGGCCAACTT
TAAGATTCGACATAACATTGAGGATGGTGGGGTACAGCTGGCGGACCACTACCAGCAAAACA
CCCCCATTGGGGACGGGCCTGTGCTCCTTCCCGACAATCATTATCTCTCCACTCAGTCAAAG
CTCTCGAAAGATCCCAATGAGAAACGGGACCATATGGTCTTGCTGGAGTTCGTCACTGCCGC
AGGCATCACACTGGGGATGGACGAGCTTTACAAG

Primers to amplify:

p30 Fw: 5'CTAGGGTTAAGCGTTGACATTGATTATTGAC3'

p30 Rv: 5'CTTCTCCTATTACTTGTAAAGCTCGTCCATC3'

SV40polyA from pMDC:

GAGAAGTACTAGAGGATCATAATCAGCCATACCACATTTGTAGAGGTTTTACTTGCTTTAAA
AAACCTCCCACACCTCCCCCTGAACCTGAAACATAAAATGAATGCAATTGTTGTTGTTAACT
TGTTTATTGCAGCTTATAATGGTTACAAATAAAGCAATAGCATCACAAATTTCACAAATAAA
GCATTTTTTTCACTGCATTCTAGTTGTGGTTTGTCCAAACTCATCAATGTATCTTATCATGT
CTGGATCTGATCACTGCTTGAGCCTAGAAGATCCGGCTGCTAACAAAGCCCGAAAGGAAGCT
GAGTTGGCTGCTGCCACCGCT

Primers to amplify:

pMDC Fw: 5’TACAAGTAATAGGAGAAGTACTAGAGGATCAT3’

pMDC Rv: 5’CTAGGGTTAAAGCGGTGGCAGCAGCCAA3’

PiggyBac 5’Terminal Repeat from pPB_CAG_EBNXN:

TTAACCCTAGAAAGATAGTCTGCGTAAAATTGACGCATGCATTCTTGAAATATTGCTCTCTC
TTTCTAAATAGCGCGAATCCGTCGCTGTGCATTTAGGACATCTCAGTCGCCGCTTGGAGCTC
CCGTGAGGCGTGCTTGTCAATGCGGTAAGTGTCACTGATTTTGAACTATAACGACCGCGTGA
GTCAAAATGACGCATGATTATCTTTTACGTGACTTTTAAGATTTAACTCATACGATAATTAT
ATTGTTATTTCATGTTCTACTTACGTGATAACTTATTATATATATATTTTCTTGTTATAGAT
ATCAACTAGAATGCTAGCATGGGCCCATC

Primers to amplify:

pPB_CAG Fw2: 5’ TGCCACCGCTTTAACCCTAGAAAGATAGTC3’

pPB_CAG Rv2: 5’ TGCCTGCCTGGATGGGCCCATGCTAGCA3’
